# Diabetes Mellitus, Mitochondrial Dysfunction and Ca^2+^-Dependent Permeability Transition Pore

**DOI:** 10.3390/ijms21186559

**Published:** 2020-09-08

**Authors:** Konstantin N. Belosludtsev, Natalia V. Belosludtseva, Mikhail V. Dubinin

**Affiliations:** 1 Department of Biochemistry, Cell Biology and Microbiology, Mari State University, pl. Lenina 1, 424001 Yoshkar-Ola, Mari El, Russia; nata.imagination@gmail.com (N.V.B.); dubinin1989@gmail.com (M.V.D.); 2 Laboratory of Mitochondrial Transport, Institute of Theoretical and Experimental Biophysics, Russian Academy of Sciences, Institutskaya 3, 142290 Pushchino, Moscow Region, Russia

**Keywords:** diabetes mellitus, mitochondria, MPT pore, mitochondrial biogenesis, mitophagy, mitochondrial dynamics, Ca^2+^ uniporter

## Abstract

Diabetes mellitus is one of the most common metabolic diseases in the developed world, and is associated either with the impaired secretion of insulin or with the resistance of cells to the actions of this hormone (type I and type II diabetes, respectively). In both cases, a common pathological change is an increase in blood glucose—hyperglycemia, which eventually can lead to serious damage to the organs and tissues of the organism. Mitochondria are one of the main targets of diabetes at the intracellular level. This review is dedicated to the analysis of recent data regarding the role of mitochondrial dysfunction in the development of diabetes mellitus. Specific areas of focus include the involvement of mitochondrial calcium transport systems and a pathophysiological phenomenon called the permeability transition pore in the pathogenesis of diabetes mellitus. The important contribution of these systems and their potential relevance as therapeutic targets in the pathology are discussed.

## 1. Introduction

Diabetes mellitus is the most common endocrine pathology in many countries around the world today. According to the data of the International Diabetes Federation for 2019, the number of people with diabetes worldwide is approximately 463 million [1]. The relevant mortality rate from diabetes in economically developed countries reaches 4% each year. The forecast of the World Health Organization by 2030 indicates that diabetes will be the seventh disease in the ranking of the causes of mortality in the world [2]. To date, diabetes is recognized as one of the concomitant diseases that causes severe complications in patients with COVID-19 [3].

Diabetes mellitus is a chronic disease that is characterized by an absolute or relative deficiency of insulin, the hormone that stimulates the transport of glucose across cell membranes, which leads to an increase in blood glucose—hyperglycemia. Respectively, two main types of diabetes are distinguished. Type I diabetes mellitus (about 10% of cases of diabetes) is an autoimmune disorder that results from the progressive destruction of the insulin-producing beta cells of the pancreas by T cells and activated macrophages and eventually leads to insulin deficiency in the organism. It is well known that type I diabetes most frequently develops in childhood and causes severe long-term complications, including retinopathy, neuropathy, and nephropathy [4,5,6]. Type II diabetes or adult- onset diabetes (about 90% of cases) is characterized by an impairment of homeostasis of glucose and insulin, in particular, the development of insulin resistance of target tissues associated with compensatory hyperinsulinemia, followed by beta-cell dysfunction. Type II diabetes mellitus is accompanied by glucose toxicity, lipotoxicity, and chronic oxidative stress, which finally can result in damage to vital organs and development of life-threatening secondary complications [4,7].

At the cellular level, diabetes mellitus, like many other metabolic abnormalities, is closely associated with alterations in the structure and function of mitochondria [8,9]. As is known, glucose is one of the primary molecules that serve as energy source in most cells and tissues of the organism. In this regard, the disorder of glucose uptake will disrupt cellular energy metabolism and, consequently, the functioning of mitochondria as a key player in the metabolism. The development of mitochondrial dysfunction in the course of diabetes mellitus was described for the first time 45 years ago [10]. Meanwhile, current data on the molecular mechanisms underlying damage to mitochondria in diabetes are rather controversial. This applies especially to a pathophysiological phenomenon called the opening of the mitochondrial permeability transition (MPT) pore, which can trigger the release of proapoptotic proteins from mitochondria and eventually result in cell death. This review focuses on the complex changes that occur in mitochondria in type I and II diabetes, as well as specific mechanisms connecting mitochondrial dysfunction and the progression of the pathology. Particular attention will be paid to the role of the Ca^2+^ handling and the MPT pore in the development of mitochondrial dysfunction in diabetes mellitus.

## 2. Mechanisms of the Diabetes-Induced Mitochondrial Dysfunction

Mitochondrial dysfunction refers to alterations in the ultrastructure and functioning of mitochondria, which can occur due to a disturbance of synthetic processes in the cell or the direct action of the damaging agents on the organelles. The crucial characteristics of mitochondrial dysfunction are decreased numbers of mitochondria in tissues, profound ultrastructural abnormalities of the organelles, impaired mitochondrial biogenesis, reduced activity of mitochondrial multienzyme complexes, and suppressed ATP synthesis. Along with other features, mitochondrial dysfunction is also characterized by the disturbance of calcium homeostasis, excessive ROS production, and the MPT pore opening, which can trigger irreversible damage to cell structures and cause apoptotic cell death. Importantly, all these processes take place in the course of type 1 and 2 diabetes mellitus, indicating mitochondrial dysfunction being implicated in the pathogenesis of the disease. The diabetes-related mitochondrial alterations in different tissues and organs of animals and humans are summarized in Table 1.

As one can see from Table 1, the rate of mitochondrial ROS production increases in most vital organs and tissues of diabetic animals and patients, while changes in mitochondrial biogenesis, dynamics, mitophagy, and oxidative phosphorylation in diabetes can be tissue-specific. Depending on the animal model used, diabetes-induced changes in some mitochondrial processes in the same tissues can be oppositely directed. Indeed, numerous animal models of diabetes are available, including genetic or spontaneously induced and experimentally induced models. Models of spontaneous type I diabetes target single or multiple genes and include a number of stains and sub-stains of rodents with genetic susceptibility for the pathology, including nonobese diabetic (NOD) mice, diabetic Bio-Breeding (BB) rats, Akita mice, Komeda Diabetes Prone (KDP) rats, LETL/KDP rats and substrains, LEW.1AR1/Ztm-iddm rats, etc. The chemical agents streptozotocin and alloxan are also commonly used to induce type I diabetes in rodents, but the regimes for their administration to animals differ significantly. Type 2 diabetes, typically accompanied by obesity, is induced by feeding a high-fat diet to animals, while diabetogenic drugs (streptozotocin, etc.) are often additionally used (for more details see [42,43,44]). Type 2 diabetes can also be produced in rodents by utilizing monogenic mutations (Lep ob/ob (*ob/ob*) mice, Lepr db/db (*db/db*) mice, Zucker diabetic fatty (ZDF-Lepr fa/fa or *fa/fa*) rats), or polygenic mutations (Kuo Kondo (KK) mice, Otsuka Long-Evans Tokushima Fat (OLEFT) rats, etc.). Models of spontaneous type II diabetes are wildly used both in a combination with obesity (Lep ob/ob (*ob/ob*) mice, etc.) and in the absence of obesity (Goto-Kakizaki (GK) rats, etc.). On the one hand, the pathophysiological and genetic features of animal models can affect the above processes in mitochondria. On the other hand, it is equally important to pay attention to the stage of development of diabetes mellitus (or the duration of its induction). For example, in humans, mitophagy in pancreatic β-cells is activated at the pre-diabetic stage, but significantly suppressed during diabetes [20]. In animal models, responses to exposure to diabetogenic drugs and high-energy diet might be the result of both adaptive and maladaptive processes, which, unfortunately, are often difficult to separate.

### 2.1. Imbalance of Mitochondrial Biogenesis and Mitophagy upon Diabetes Mellitus

The maintenance of the content and structure of mitochondria in the cell (mitochondrial homeostasis) is ensured by the coordination of two opposite processes—mitochondrial biogenesis and mitophagy.

Mitochondrial biogenesis is a complex and highly regulated process that requires the coordinated cooperation of transcription and replication of both the nuclear and the mitochondrial genomes. The master regulator that provides the transcriptional control of mitochondrial biogenesis is the peroxisome proliferator-activated receptor γ (PPARγ) coactivator-1α (PGC-1α) [45]. The expression of PGC-1α can be regulated by many extra and intracellular signals mediated by CREB, AMP-activated protein kinase (AMPK), Ca^2+^/calmodulin-dependent protein kinase (CAMK), calcineurin, and nitric oxide (NO) [45,46,47,48,49,50]. PGC-1α coactivates a number of transcription factors, including nuclear respiratory factor 1 (NRF1), estrogen-related receptors (ERRs), and PPARs. In turn, these factors activate the expression of the nuclear genes encoding almost all mitochondrial proteins. In addition, PGC-1α was also shown to promotes the expression of two isoforms of a mitochondrial transcription specificity factor, termed TFA1M and TFB2M, and the mitochondrial DNA polymerase, which induces the transcription and replication of mitochondrial DNA (mtDNA). The role of PGC-1α in regulating cellular metabolism was described in detail in recent reviews [45,51,52].

The evidence supports that the diabetes-related suppression of energy metabolism is often associated with a decrease in the content of mitochondria and the impairment of mitochondrial biogenesis in damaged cells [53,54,55]. Indeed, the expression levels of PGC-1α, mitochondrial proteins and mRNA, as well the ratio between the number of copies of mtDNA and nuclear DNA (nDNA) are reduced in many tissues and vital organs (in particular, the cardiac and skeletal muscles, the kidneys, and the brain) of both humans and laboratory animals with diabetes (Table 1). The administration of insulin to animals with type I diabetes leads to an increase in the expression level of mitochondrial proteins and an improvement of energy metabolism (an increase in the rate of ATP synthesis) [56,57]. The use of antidiabetic drugs (metformin, thiazolidinediones, empagliflozin, and some others) or regular exercise recovers the expression of PGC-1α, the mtDNA/nDNA ratio and improves mitochondrial energy metabolism in diabetic animals [58,59,60,61,62]. At the same time, an increase in the expression of PGC-1α results in the restoration of both energy functions and insulin sensitivity of cells [62,63].

Interestingly, the expression level of PGC-1α in hepatocytes can increase in diabetic conditions, leading to an elevation of the production of glucose in the liver and the aggravation of hyperglycemia [19]. The knockdown of PGC-1α in the liver tissue improves glucose tolerance and hepatic insulin sensitivity in *db/db* mice [64].

Meanwhile, some studies have shown that the number of mitochondria in some cells does not change or even increases in the course of diabetes. It was found that in patients with type I and type II diabetes, there is no decrease in the number of mitochondria in the pancreas [65]. In cardiomyocytes, the mtDNA/nDNA ratio, the size and number of mitochondria upon diabetes type I and II are elevated [13]. In parallel, an increase in the production of reactive oxygen species and a suppression of the function of the mitochondrial respiratory chain are observed [66,67]. One can suggest that in these studies, adaptative changes occurred. In this case, increased mitochondrial biogenesis could be a response to mitochondrial damage due to oxidative stress. Together with the suppression of mitophagy, this may lead to an increase in the number of mitochondria (including the damaged ones) in cells of some tissues upon diabetes.

Mitophagy is the process of selective destruction of mitochondria by autophagy. The mechanism is essential for a living cell to maintain mitochondrial quality and homeostasis by removing the needless or dysfunctional mitochondria. Mitophagy also protects against apoptosis and alleviates cell damage from toxic substances [68]. The stimulation of mitophagy can occur due to the activation of sirtuin-1 (SIRT1), NAD^+^-dependent histone/protein deacetylase, and AMPK, which, in turn, initiates two signaling pathways containing PTEN-induced putative kinase 1 (PINK1), the E3 ubiquitin ligase Parkin (PINK1/Parkin), and NIP3-like protein X (NIX)/BNIP3. These signaling mechanisms are triggered in the cell in response to the occurrence of damaged mitochondria (for example, mitochondria with low membrane potential), which leads to the formation of autophagosomes that subsequently deliver them to lysosomes for complete destruction [69]. It is the balance between mitochondrial biogenesis and mitophagy that maintains a constant number of functionally active mitochondria in the cell. Therefore, these processes can be triggered when specific signaling pathways are activated.

The expression of PINK1, Parkin, NIX, and other autophagy-related proteins was found to increase in tissues of patients with a pre-diabetic state [20]. In rodent models of type 2 diabetes induced by a high-fat diet and low doses of streptozotocin treatment, autophagy was shown to be activated in pancreatic β-cells [21,70]. With the progression of diabetes mellitus in humans and laboratory animals, the mitochondrial-specific autophagy is suppressed in many tissues and organs (pancreas, heart, skeletal muscle, eyes) [20,22,23,24,25,28,71], which may be associated with a decrease in the activation of AMPK and SIRT1 [72,73,74]. Some studies revealed that the chronically elevated level of glucose upon type I diabetes induces the accumulation of p53 protein in the cytoplasm of β-cells of the pancreas. This protein binds to Parkin, blocks its translocation to damaged mitochondria and thereby inhibits mitophagy [75]. There is evidence that anti-diabetic drugs can enhance the autophagic clearance of damaged mitochondria, which leads to the improvement of mitochondrial energetics. It was observed that metformin operates as an agonist of AMPK (and SIRT1) and increases the expression of Parkin [25,76], whereas the inhibitors of sodium-glucose linked transporter 2 (SGLT2) activate the SIRT signaling pathway and AMPK, thereby promoting mitophagy [77,78].

**Figure 1:**
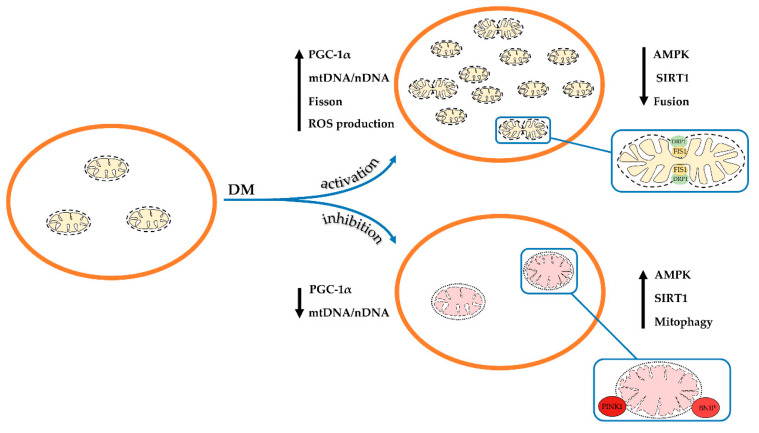
Schematic illustration of changes in the ultrastructure and content and of mitochondria in the cell in diabetes mellitus. Depending on the model of diabetes used or tissue, mitochondrial biogenesis and mitophagy can be enhanced or suppressed. The arrows show changes in the content of mitochondrial DNA, the main proteins responsible for mitochondrial dynamics, and the rate of ROS production by mitochondria in the pathology.

Thus, one can conclude that the development and progression of diabetes and its complications in humans and animals mainly leads to the suppression of both mitochondrial biogenesis and mitophagy. As a result, the number of healthy mitochondria in the cell decreases and the proportion of the depolarized organelles increases. These changes, obviously, underlie overall mitochondrial dysfunction and abnormality throughout diabetes, which will be discussed below. At the same time, a number of studies showed the stimulation of the processes of mitochondrial biogenesis and mitophagy (Figure 1). These observations suggest the occurrence of adaptive changes in various tissues, which may prevent or delay the onset of diabetes-related complications. In this regard, further research is required to elucidate the mechanisms of changes in mitochondria at different stages in the disease progression.

### 2.2. Impairment of Mitochondrial Dynamics in Diabetes Mellitus

Mitochondrial homeostasis in the cell is supported not only by the processes of mitophagy and biogenesis but also by many other mechanisms of mitochondrial quality control. Changes in the architecture of the mitochondrial network due to repeated fusion and fission of the organelles is another mechanism that regulates the maintenance of proper mitochondrial functions. Mitochondria are remarkably dynamic organelles; their shape, position in the cell, arrangement of the cristae, and other morphological signs can vary significantly depending on physiological or pathological conditions. It has been suggested that conditions that require increased energy expenditure (fasting, acute stress, and others) promote mitochondrial fusion and the formation of an interconnected/elongated mitochondrial network. Conversely, conditions of excess energy sources and relatively low demand for ATP stimulate mitochondrial division and fragmentation of the mitochondrial network, which results in an increase in the proportion of single mitochondria in the cell. In addition, mitochondrial fragmentation has been thought to be essential for mitophagy induction [79,80].

The dynamic balance between fission and fusion is regulated by large GTPase family proteins belonging to the Dynamin superfamily. Mitochondrial fission is mainly ensured by dynamin-related protein 1 (DRP1) and fission protein 1 (FIS1) [79,81]. DRP1 mediates mitochondrial construction by assembling into an oligomeric ring in the constriction sites, which divides the mitochondrion in a GTP-dependent process. Drp1 is primarily localized in the cytoplasm, but it can be recruited to mitochondria via receptors anchored into the outer membrane of the organelles. FIS1, a small adaptor protein located in the outer mitochondrial membrane, participates in the recruitment of DRP1 through its cytosolic domain. Along with FIS1 and DRP1, three more proteins have been identified to be involved in mitochondrial fission: mitochondrial dynamics proteins of 49 and 51 kDa (MiD49 and MiD51, respectively) and mitochondrial fission factor (MFF) [82].

The fusion of the outer mitochondrial membranes is mediated by mitofusins 1 and 2 (MFN1 and MFN2) [79,81]. Mfn2 is also one of the linker proteins that is involved in the formation of contact sites between mitochondria and the endoplasmic reticulum (ER), termed mitochondria-associated ER membranes (MAMs). MAMs play a fundamental role in the regulation of cellular metabolism and, in particular, glucose and Ca^2+^ homeostasis [83,84]. The fusion of the inner mitochondrial membranes is carried out by optic atrophy 1 (OPA1) (see reviews [79,81,85]). OPA1 has a dual role: it is also involved in maintaining the structure of mitochondrial cristae and thereby can modulate mitochondrial function through cristae remodeling [86].

There is increasing evidence that altered insulin signaling in cells may contribute to changes in the level of proteins responsible for mitochondrial fusion and in the structure of the mitochondrial network. It has been shown that the expression of MFN2and OPA1 is significantly decreased in many tissues of diabetic animals [29,30,32,33,36,87,88,89]. Treatment with the mitochondrial fusion promoter-M1 increases the expression of OPA1, promotes mitochondrial fusion, enhances mitochondrial respiratory capacity, and reduces the rate of ROS production by mitochondria [90]. At the same time, the function of the mitochondrial fission machinery in diabetes is enhanced. In particular, increased expression of DRP1 and FIS1 is observed [28,30,31,32,33,34,87,89]. In cultured skeletal muscle cells and cortical neurons, genetic and pharmacological inhibition of Drp1 was shown to attenuate palmitate-induced mitochondrial fragmentation and insulin resistance [91,92]. These events were accompanied by a decline in ROS generation by mitochondria and an increase in the mitochondrial respiratory capacity.

As mentioned above, mitochondrial fragmentation can play an important role in the induction of mitophagy in the cell. At the same time, there is evidence that DRP1 is not essential for mitophagy, but rather restricts PINK1–Parkin activity to specific mitochondrial subdomains. In this regard, one can assume that an increase in DRP1 in diabetes can lead to suppression of mitophagy [93].

Along with the enhanced fission and fragmentation of mitochondria in diabetes mellitus, pronounced alterations in the ultrastructure of the organelles are observed. Transmission electron microscopy analysis reveals the occurrence of swollen “hypertrophic” mitochondria with a decreased matrix density and disorganized inner-membrane cristae in cells from various tissues and organs [29,88].

It was found that antidiabetic drugs can restore mitochondrial fusion and fission within the cells. Notably, the SGLT2 inhibitors empagliflozin and dapagliflozin modulate the activity of mitochondrial dynamics via the regulation of fission (FIS1 and DRP1) and fusion (MFN1 and MFN2) proteins [34,94,95]. Metformin reduces the expression of DRP1 [96]. It was also demonstrated that exercise promotes a decrease in DRP1 expression and an increase in MFN1 and MFN2 expression. The dipeptidyl peptidase-4 inhibitor vildagliptin suppresses the expression of FIS1 and DRP1 and prevents the translocation of DRP1 into mitochondria [97].

Thus, one can conclude that during diabetes mellitus, the system of mitochondrial fission and fusion is thrown out of balance, which leads to the remodeling of the mitochondrial network in the cell. Ultimately, this is accompanied by a change in the structure of mitochondria and their functional activity (Figure 1).

### 2.3. Diabetes-Induced Changes in the Functional Activity of Mitochondria

In the course of both type 1 and 2 diabetes mellitus, abnormal glucose utilization leads to a “switch” of energy metabolism: the cells begin to replenish their energy needs mainly via fatty acid β-oxidation. Although the mechanisms by which this occurs are different for the two types of diabetes, there is eventually an increased uptake and utilization of fatty acids in both cases. An increase in the plasma level of free fatty acids and their uptake by cells in type II diabetes further reduces the insulin-dependent absorption of glucose. In parallel, high rate of gluconeogenesis is observed [98,99]. Ultimately, this results in the fact that the synthesis of ATP in cells occurs mainly due to the oxidation of fatty acids, and not the metabolism of carbohydrates [54].

In parallel, there is a PPARα-mediated increase in the expression of mitochondrial enzymes responsible for the metabolism of fatty acids, notably carnitine palmitoyl transferase 1 (CPT1) [100]. Inhibition of CPT1 is considered as a possible mechanism to modulate altered energy metabolism and improve insulin sensitivity of cells upon type II diabetes [101].

As is known, type II diabetes leads to serious disorders of protein metabolism. In mitochondria, the inhibition of pyruvate dehydrogenase, branched-chain a-ketoacid dehydrogenase, and tyrosine aminotransferase occurs. These events would selectively increase tissue and blood concentrations of some essential amino acids, particularly methionine [102]. In this regard, methionine restriction can improve insulin resistance and glucose homeostasis in diabetes [103].

As would be expected, changes in the cellular energy metabolism and structure of mitochondria in type I and II diabetes, should lead to impaired mitochondrial respiration and oxidative phosphorylation. Meanwhile, the literature data are rather contradictory.

On the one hand, some studies demonstrated that respiratory function and ATP synthesis in diabetic mitochondria are suppressed. It was found that the ADP/O ratio and the rate of ADP-stimulated respiration of isolated mitochondria harvested from diabetic patients and different animal models for diabetes significantly decrease [104,105,106]. These events are accompanied by an increase in the NADH/NAD ratio due to a pronounced dysfunction of complex I of the mitochondrial respiratory chain, as well as a decrease in the activity of complexes II, IV, and V (ATP synthase) [13,107]. The content of the ATP synthase complex in mitochondria of the liver and the heart of animals with type 1 diabetes is also reduced, which can be associated with the accumulation of calpain 1 in the organelles [41,108]. The alterations in activities and protein expression of the respiratory chain complexes both inhibit ATP synthesis and accelerate the generation of ROS in mitochondria, resulting in the development of oxidative stress. Inhibition of calpain 1 was shown to restore the levels of ATP synthase-α (*ATP5A1*) and autophagy, as well as prevent mitochondrial fragmentation and excessive ROS production [108,109].

It is known that the individual complexes of the mitochondrial respiratory chain are capable of organizing into multienzyme assemblies, so-called supercomplexes, which increases the efficiency of ATP synthesis. Destabilization of the structure of the mitochondrial supercomplexes might contribute to the disruption of mitochondrial respiration and oxidative phosphorylation. In tissues of patients with type 2 diabetes, the extent of assembly of the respiratory supercomplex I-III-IV was found to decrease compared with that in the control [110]. Similar changes were demonstrated in diabetic mice that were fed with a high cholesterol diet. In these animals, the rate of mitochondrial state 3 respiration, the value of the mitochondrial membrane potential, and the assembly of the respiratory supercomplexes in mitochondria of the liver were reduced [111]. Some studies also revealed that a diabetes-induced decline in the activity of complexes of the respiratory chain can be associated with a decreased content or altered composition (peroxidation) of cardiolipin, a main anionic phospholipid in the inner mitochondrial membrane (see review [54]).

On the other hand, several studies showed that diabetes is not accompanied by a significant decrease in the ability of the mitochondria to synthesize ATP. The studies were performed on mitochondria isolated from various tissues of patients with both type 1 and type 2 diabetes and some diet-induced and transgenic diabetic animal models (see review [37]). Mitochondria respiration in these experiments was supported by oxidation of substrates of complexes I and II of the respiratory chain [112,113]. In some cases, a diabetes-induced stimulation of respiration of mitochondria was even observed. For example, mitochondrial respiration was found to increase in the diabetic liver, where the level of cardiolipin in the mitochondrial membranes was also elevated [113]. Therefore, an increase in the phospholipid level in diabetes is likely to be required for adaptive responses, whereas a loss of its content in mitochondria can contribute to the dysfunction of the organelles [114].

It should be noted that some antidiabetic drugs can suppress oxidative phosphorylation of isolated mitochondria [115]. Metformin and pioglitazone inhibit the activity of complex I of the mitochondrial respiratory chain [115,116,117]. Pioglitazone at high concentrations induces mitochondrial swelling, increases ROS production, and decreases the membrane potential of mitochondria [118].

As mentioned above, the development of diabetes is accompanied by the accumulation of free fatty acids inside cells and intracellular membranes. In mitochondria, this can lead to uncoupling of respiration and oxidative phosphorylation as well as decreased ATP production [66]. The uncoupling action of free fatty acids on oxidative phosphorylation is carried out by the protonophore mechanism and mediated by anion carriers of the inner mitochondrial membrane: uncoupling proteins (UCP1-3), adenine nucleotide translocator (ANT), aspartate/glutamate carrier, etc. Despite the seemingly negative events, uncoupling of oxidative phosphorylation in mitochondria is of great physiological significance. Especially, mitochondrial uncoupling triggers nonshivering thermogenesis in brown adipose tissue and reduces the excessive generation of ROS by mitochondria [66,119,120].

Some studies reported that the expression of uncoupling proteins (UCPs) 2 and 3, which mediate proton leak across the inner mitochondrial membrane when activated by fatty acids, is increased in many tissues of animals with experimental diabetes [66,121,122,123]. Further investigations in this field revealed that UCPs may be involved in the development of mitochondrial dysfunction in diabetes, and their impact on mitochondrial metabolism is dependent on the used model of diabetes mellitus. It was found that after a long-term high-fat diet in animals, UCP3 induces mitochondrial uncoupling and reduces cardiac efficiency. Meanwhile, UCP3 does not mediate mitochondrial uncoupling in leptin-deficient states of animals (*ob/ob* mice). It should be noted that in the latter case, an increase in UCP expression may be an adaptive mechanism in response to an excessive ROS generation by mitochondria [124]. Studies suggested that it is the prevention of ROS overproduction and ROS- induced cell death that is the main result of an increase in the content of this protein in the mitochondrial membrane. So, overexpression of UCP2 was found to inhibit ROS generation and high glucose-induced apoptosis of human umbilical vein endothelial cells [122].

### 2.4. Oxidative Stress and Diabetes Mellitus

Oxidative stress is a key component in the pathogenesis of diabetes mellitus and the only pathogenic factor that almost all available studies point to [13,54,125]. Only a small number of studies indicate that mitochondrial dysfunction in diabetes is not necessarily linked with an overproduction of ROS [126,127]. Except for a few papers, most studies demonstrate that the generation of ROS by mitochondria is increased dramatically in many tissues and metabolically active organs in obesity and insulin resistance [17,20,34,38,39,40,41]. Moreover, mitochondrial ROS modulates various pathophysiological processes in cells upon diabetes, ranging from an adaptive metabolic response to ROS production and ending with ROS-induced cell death [54,125].

The main resource of ROS in mitochondria is the electron transport chain, namely, complexes I (NADH-ubiquinone oxidoreductase) and III (ubiquinol-cytochrome *c* oxidoreductase). In these complexes, molecular oxygen is reduced to superoxide anion radical (O_2_^−^). Superoxide can also be formed during reverse electron transfer from ubiquinol to complex I in an over-reduced electron transport chain, which leads to the reduction of NAD to NADH. Being short-lived, superoxide can spontaneously or enzymatically convert to H_2_O_2_. In turn, H_2_O_2_ and O_2_^−^ can be further converted to the extremely active hydroxyl radical (OH) in the Fenton reaction. Maintaining a low level of harmful reactive radicals in cells is provided by the antioxidant defense system, which includes the enzymatic proteins manganese superoxide dismutase (MnSOD), catalase, glutathione peroxidase, thioredoxins, peroxiredoxins, as well as non-enzymatic scavengers such as glutathione, tocopherol, and others (see reviews [128,129]).

The association of an overproduction of ROS by mitochondria with the occurrence of cell resistance to insulin has been proven convincingly. This perspective was detailed in the review by Yaribeiga and others who considered five main molecular mechanisms through which oxidative stress induces insulin resistance: β-cell dysfunction, decreased expression of the glucose transporter GLUT4, suppression of insulin signaling pathways, increased inflammatory responses, and mitochondrial dysfunction [125].

It is commonly accepted that elevated ROS levels can suppress ATP synthesis in mitochondria. First, ROS can react with the unsaturated fatty acids of lipids of mitochondrial membranes and induce lipid peroxidation. The oxidative degradation of membrane phospholipids may promote non-specific permeabilization of the inner mitochondrial membrane, increase proton leakage, and inhibit the activity of respiratory chain complexes. As mentioned above, the peroxidation of cardiolipin results in the dysfunction of complex I and destabilization of supercomplex assemblies in the mitochondrial membranes during diabetes [54]. Second, suppression of oxidative phosphorylation by ROS may be associated with oxidative damage or decreased level of mitochondrial sirtuin 3 (SIRT3), a major NAD-dependent protein deacetylase [107,130]. In healthy cells, SIRT3 plays a key role in deacetylating and modifying the enzymatic activities of several mitochondrial proteins including the respiratory chain complexes I, II, and III [130]. It was shown that SIRT3 protein level is significantly reduced in the tissues of mice with type 1 and 2 diabetes [131]. In *Sirt3* knock-out mice, the hyperacetylation of the mitochondrial respiratory chain complexes I and III is associated with the development of oxidative stress in cells [22,107,130]. Furthermore, these events are accompanied by the suppression of mitochondrial respiration and inactivation of the primary antioxidant enzyme MnSOD [130,132]. Third, elevated ROS formation is one mechanism of upregulation of UCPs, which eventually may lead to a decrease in mitochondria-generated ATP levels in cells [54]. Fourth, ROS-induced oxidative damage to mitochondrial proteins can disturb their functions and trigger the opening of the Ca^2+^-dependent mitochondrial permeability transition (MPT) pore. The pathological phenomenon of the MPT pore opening is characterized by a sudden loss of permeability of the inner mitochondrial membrane, which can lead to the collapse of the membrane potential, release of proapoptotic proteins from mitochondria, and ultimately cell death [133]. Finally, excessive ROS production can induce oxidative damage to mtDNA followed by deficiencies of the respiratory chain complexes and total mitochondrial dysfunction [134].

Interestingly, ROS can be an activator of autophagy/mitophagy [135]. It can be suggested that, like upregulation of mitochondrial uncoupling proteins, this mechanism works in pre-diabetic or early stages of diabetes mellitus. However, in the late stages of diabetes, when mitophagy is suppressed, this compensatory mechanism is apparently suppressed [20].

Recent studies suggest that treatment with metformin, thiazolidinediones, SGLT2 and DPP4 inhibitors prevents both the development of oxidative stress and mitochondrial dysfunction in diabetes [34,96,97,136,137,138]. A growing body of evidence suggests that hypoglycemic drugs eliminate most of the signs of mitochondrial dysfunction in cells of various tissues and organs of diabetic animals.

The use of regulators of oxidative stress in the comprehensive treatment of diabetes was found to maintain the structural and functional integrity of mitochondria. It has been shown that the mitochondrial-targeted antioxidants MitoTEMPO, MitoQ, BAM15, C12TPP, and CoQ10 partially prevent the ROS-induced disorders in oxidative phosphorylation and ultrastructure of mitochondria in animal models of obesity and insulin resistance [38,139,140,141,142]. Currently, the positive effects of CoQ10 and resveratrol as antidiabetic drugs have been demonstrated in most clinical trials [143]. At the same time, the administration of antioxidants has been reported to usually attenuate diabetes-associated pathological changes in cells, but not always affect glucose levels and insulin sensitivity of the organism (see review [143]).

The development and progression of oxidative stress in animal models of diabetes can also be regulated by modulating the expression and activity of major enzymes responsible for antioxidant protection. It has been shown that overexpression of catalase in muscle mitochondria of obese mice leads to the improvement of insulin sensitivity of the animals fed with a high-fat diet [144]. Increased expression of peroxiredoxin 3 or MnSOD was found to preserve cardiomyocytes and prevent the development of diabetic cardiomyopathy [145,146].

The free radical overload is one of the most common features of damaged mitochondria in diabetes mellitus. In addition to the above effects, elevated ROS levels in mitochondria dysregulates Ca^2+^ homeostasis through the induction of Ca^2+^ overload and the Ca^2+^-dependent formation of the MPT pore. Even though ROS are traditionally considered as one of the main activators of the mitochondrial pore, the data on the involvement of the MPT pore opening in the pathology of diabetes mellitus are currently highly controversial. In the next section, we attempt to summarize the recent data on the role of the MPT pore in the development of diabetes-related mitochondrial dysfunction and to establish the association between this pathological phenomenon in mitochondria and diabetes mellitus.

## 3. Ca^2+^ Handling, MPT Pore and Diabetes Mellitus

### 3.1. Mitochondrial Ca^2+^ Transport in Diabetes

Ca^2+^ is a universal regulator of many intracellular processes. The appearance of Ca^2+^ in the cytosol of pancreatic β-cells is one of the important steps in the mechanism of insulin secretion and regulation of glucose metabolism in humans and animals [147]. On the other hand, higher intracellular Ca^2+^ level has been found in primary adipocytes, hepatocytes, and cardiomyocytes isolated from obese human subjects with insulin resistance as well as diabetic animals [148,149,150]. In this regard, maintaining a low Ca^2+^ concentration inside cells is an important function of a number of structures, including mitochondria. Activation of mitochondrial Ca^2+^ uptake occurs at a sufficiently high ion concentration in the cytosol. Mitochondria have the ability to rapidly transport and store high concentrations of Ca^2+^ in the matrix, which is extremely important for the regulation of calcium homeostasis under stressful conditions. Mitochondrial matrix Ca^2+^ regulates a fairly wide range of proteins of the tricarboxylic acid cycle (in particular, pyruvate dehydrogenase, citrate dehydrogenase, and α-ketoglutarate dehydrogenase), respiratory chain complexes, contributing to the maintenance of cell energy metabolism, and ATP generation. It should also be noted that excessive accumulation of Ca^2+^ in mitochondria leads to the opening of the MPT pore in the inner membrane and cell death initiation [84,151].

Ca^2+^ enters the mitochondria through the voltage-dependent anion channel (VDAC) located on the outer mitochondrial membrane. VDAC is one of the components of the MAM (mitochondria-associated membrane) contacts, which allows Ca^2+^ released from the endoplasmic reticulum to be immediately redirected via IP_3_ receptors into mitochondria. The main component responsible for mitochondrial Ca^2+^ handling is the Ca^2+^ uniporter of the inner mitochondrial membrane. The uniporter has a remarkably high affinity for Ca^2+^, since other divalent cations are transported inside the mitochondria much more slowly and, moreover, inhibit Ca^2+^ uptake. It is inhibited by the polycationic dye ruthenium red and its analogues. Nowadays it is recognized that the mitochondrial calcium uniporter is a multicomponent system—the pore channel is formed by MCU integral membrane proteins (there is also an inactive MCU paralogue—MCUb), Ca^2+^ uptake is regulated by MICU (MICU1–3) gate proteins, as well as regulatory proteins EMRE and MCUR1. It is important to note that the level of these proteins varies in different tissues, and the ratio of regulatory and channel subunits determines the ability of the mitochondria of a particular tissue to absorb calcium ions. Along with the mitochondrial uniporter, other structures are considered as possible Ca^2+^ uptake mechanisms: the rapid mode of uptake or RaM, the mitochondrial ryanodine receptor, and the Ca^2+^/H^+^ exchanger Letm1. However, their contribution to mitochondrial Ca^2+^ uptake is not as significant compared to the Ca^2+^ uniporter. The structures responsible for the release of Ca^2+^ from mitochondria include Na^+^/Ca^2+^ and H^+^/Ca^2+^ exchanges. These systems are supposed to function in different tissues: Na^+^/Ca^2+^ exchange takes place in excitable tissues, while H^+^/Ca^2+^ exchange occurs in non-excitable tissues. It has been shown that these are systems of slow release of Ca^2+^ from mitochondria, the rate of ion transport through them is much lower than the rate of Ca^2+^ entry through the Ca^2+^ uniporter. The carrier responsible for the Na^+^/Ca^2+^ exchange is an antiporter of the inner mitochondrial membrane, capable of releasing Ca^2+^ in exchange for Na^+^ or Li^+^ (NCLX— Na^+^/Li^+^/Ca^2+^ exchanger). It is assumed that Letm1 functions as a Ca^2+^/2H^+^ exchanger. In addition to these systems of slow release of Ca^2+^ from mitochondria, a sharp rapid discharge of mitochondria from Ca^2+^ occurs by the opening of non-specific Ca^2+^-dependent mitochondrial pores. The balance between mitochondrial calcium entry and release is responsible for the maintenance of intracellular Ca^2+^ homeostasis under normal and pathological conditions. More details about mitochondrial Ca^2+^ transport systems are described in reviews [84,133,151,152].

Intramitochondrial Ca^2+^ is shown to be involved in the regulation of insulin secretion in pancreatic β-cells under normal conditions [147]. The intake of glucose in β-cells leads to the accumulation of Ca^2+^ in the mitochondria, an increase in the concentration of ATP in the cells, and insulin secretion. Indeed, MCU knockout mice show inhibition of the first phase of insulin secretion [153]. It should also be noted that glucolipotoxicity is associated with suppression of Ca^2+^ transport in the mitochondria of β-cells and an increase in the level of ATP in the cytosol. However, the expression of MCU and NCLX did not change. The authors suggested that the dysregulation of Ca^2+^ transport in the mitochondria of β-cells under glucolipotoxicity is due to a change in the ultrastructure of organelles [154]. At the same time, palmitic acid upregulated MCU protein expression in mouse clonal β-cell MIN6 under normal glucose, but not high glucose medium. The authors suggested that high glucose attenuates the compensatory mechanism involving MCU in palmitate-induced cytotoxicity and causes further serious consequences related to Ca^2+^ overload in β-cell lipotoxicity [155].

One of the diabetes-related pathological changes in β-cells is the development of ER stress [156]. Along with the suppression of Ca^2+^ transport in mitochondria, this causes an increase in free Ca^2+^ in the cytoplasm, which, in turn, can lead to an imbalance of diverse Ca^2+^-dependent signaling pathways. In addition, the induction of mitochondrial dysfunction and ER stress in the pancreas may eventually result in the death of β-cells and an increase in diabetic complication rates.

**Figure 2:**
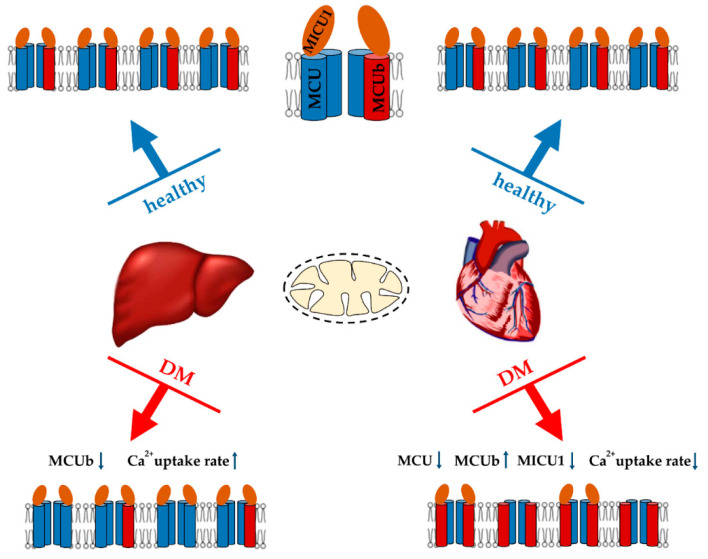
Major changes in the Ca^2+^ uniport system of liver and heart mitochondria in streptozotocin-induced diabetes mellitus (T1DM) and their effect on the Ca^2+^ uptake by organelles. An increase or decrease in the level of MCUC subunits, as well as in Ca^2+^ uptake rate is indicated by arrows (↑ or ↓).

Data on the functioning of the calcium uniporter in diabetic tissues are quite contradictory (Figure 2). A decrease in the rate of Ca^2+^ transport was observed in heart mitochondria in streptozotocin- induced T1DM rats and in T2DM *ob/ob* mice, as well as in pancreatic cells [154,157,158,159]. Heart mitochondria also showed a decrease in MCU expression in a murine model of streptozotocin-induced T1DM, which was accompanied by a suppression of mitochondrial Ca^2+^ uptake [160]. An elevated level of the dominant negative MCUb subunit of the uniporter is also expected to contribute to this picture. Indeed, normalization of the MCU level in hearts restored mitochondrial Ca^2+^ handling, increased pyruvate dehydrogenase activity, and reprogrammed a metabolism toward normal glucose oxidation [160,161]. In addition, the heart of *db/db* mice showed reduced expression of the peripheral membrane MiCU1 protein acting as a gatekeeper. The reconstitution of MiCU1 in diabetic hearts significantly inhibited the development of diabetic cardiomyopathy by increasing mitochondrial Ca^2+^ uptake and subsequently activating the antioxidant system [162].

On the other hand, there is evidence that the development of diabetes is accompanied by activation of the calcium uniporter. Indeed, as far back as the 70s, it was shown that the induction of alloxan diabetes stimulates the entry of Ca^2+^ into liver mitochondria [163]. Recently, we also demonstrated that two weeks after streptozotocin administration to Sprague-Dawley rats, the rate of Ca^2+^ uptake by liver mitochondria significantly increased. The analysis showed that an increase in the Ca^2+^ transport rate was due to a decrease in the expression of the dominant-negative MCUb subunit of the uniporter [41]. Adipose tissue mitochondria also show an increase in Ca^2+^ handling. It was shown that mitochondrial Ca^2+^ uptake increased and MCU components (MCU and MICU1) were upregulated in insulin-resistant adipocytes. Similar results were observed in mouse (*db/db* and *ob/ob*) and human visceral adipose tissue during the progression of obesity and diabetes [164].

The development of diabetes mellitus changes not only the activity and expression of Ca^2+^ uniporter, but also NCLX. Indeed, the endothelia of streptozotocin-induced T1DM rats demonstrated an increase in NCLX expression. In this case, silencing of NCLX expression increased ROS generation and NLRP3 inflammasome activation [165].

Insulin resistance and T2DM cause a disruption in the structure of MAM contacts [157,166,167,168]. The antidiabetic drugs metformin and rosiglitazone restore the structure of MAM contacts in diabetic animals [168]. It should be noted that diabetes mellitus is associated with overexpression of VDAC1 in certain tissues (pancreatic β-cells, vascular endothelial cells) [169,170,171]. In parallel, an increased amount of Ca^2+^ accumulates in mitochondria, which ultimately leads to the activation of apoptosis. Inhibition of VDAC1 overexpression leads to the suppression of apoptosis in endothelial cells and improves insulin secretion in islets [170,171].

The contradictions observed in studies of mitochondrial Ca^2+^ transport in diabetes mellitus are difficult to explain. It is possible that the development of diabetes shows tissue specificity. As mentioned above, liver cells react differently to diabetes. In particular, this organ shows PGC1-1α overexpression and stimulation of biogenesis. It is worth noting that similar adaptive changes may possibly occur in other tissues in the early stages of diabetes. Several studies on the induction of diabetes have shown an increase in the concentration of Ca^2+^ in the cytosol and mitochondria. In this regard, it can be speculated that under these conditions, the observed activation of Ca^2+^ uptake and release systems from mitochondria will lead to Ca^2+^ recyclization through the mitochondrial membrane (futile cycle) and ΔΨ decrease. Like UCP expression, this adaptive reaction will suppress oxidative stress, at least in the initial stages of the development of the disease. Such a futile cycle, causing a slight depolarization, is expected to stimulate mitophagy. Along with increased biogenesis, this will trigger the renewal of the mitochondrial population in the cell. Meanwhile, excessive accumulation of Ca^2+^ in mitochondria will undoubtedly cause the opening of the MPT pore and cell death initiation.

### 3.2. Mitochondrial Permeability Transition Pore

Excessive accumulation of Ca^2+^ in the mitochondrial matrix is known to lead to an abrupt increase in nonspecific permeability of the inner mitochondrial membrane (referred to as the mitochondrial permeability transition (MPT) pore) for various ions and hydrophilic compounds with a molecular weight of up to 1.5 kDa. This leads to swelling of the mitochondria, equilibration of ionic gradients across the inner membrane, a decrease in the mitochondrial membrane potential, and impaired ATP synthesis. The final consequence of the opening of the MPT pore is cell death. Ca^2+^-dependent permeabilization of the inner mitochondrial membrane is one of the key elements in the process of cell death during hypoxia and subsequent reoxygenation. Moreover, convincing evidence has accumulated over the years supporting an essential role of the MPT pore opening in the development of cardiovascular diseases, neurodegenerative processes, viral diseases, muscular dystrophies, etc. [84,172,173,174,175].

By the mid-90s of the last century, most of the modulators of the MPT pore had been elucidated. This is described in detail in a review by Zoratti and Szabo. Ca^2+^ is perhaps the main pore activator. In addition to Ca^2+^, inorganic phosphate (and polyphosphates), SH-oxidizing agents, oxidative stress, uncouplers, a decrease of the mitochondrial adenine nucleotide content, and other factors stimulate MPT pore opening. Inhibitors of the mitochondrial pore are the cyclosporins (cyclosporin A is most effective), adenine nucleotides, SH-reducing agents, reduced pyridine nucleotides, etc. [175].

**Figure 3:**
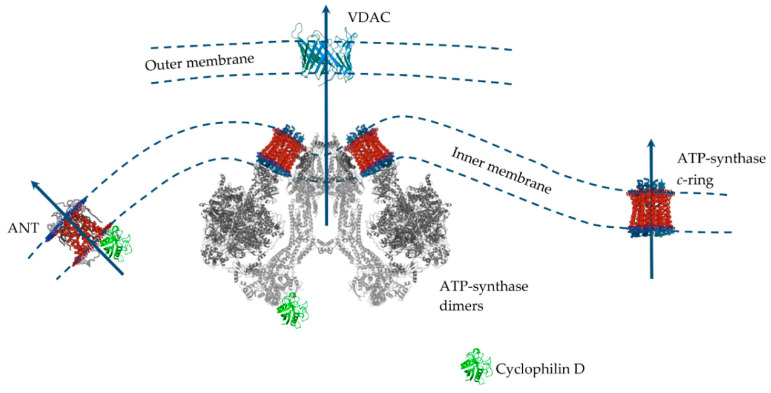
The putative MPT pore components of the inner and outer mitochondrial membranes. Proteins are drawn as ribbon representations (modified PDB ID codes: 6RD4 (F-ATP synthase); 5CBV (Cyclophilin D); 2JK4 (VDAC); 1OKC (ANT).

Despite significant progress in the study of MPT pore induction and regulation, its molecular structure and protein composition are still under discussion (Figure 3). An analysis of the literature data suggests that the MPT pore is a nonselective, high-conductance megachannel consisting of proteins of the inner and outer mitochondrial membranes. However, to date, cyclophilin D is the only, precisely established component of this structure; it is the pharmacological target of cyclosporin A and its analogues, which can specifically block pore opening [176,177]. Cyclophilin D is considered as a regulatory protein, which in the presence of Ca^2+^ stimulates rearrangement in the proteins responsible for the formation of the MPT pore channel. Knockout of cyclophilin D or its binding to an inhibitor leads to a significant increase in the threshold concentration of Ca^2+^ necessary for the pore opening. In 2015, using RNAi-based screening, it was suggested that, along with cyclophilin D, spastic paraplegia 7 (SPG7) is an important regulatory component of MPT [176]. However, it has recently been shown that SPG7 does not constitute a core component of MPT, but instead regulates activity by lowering the basal mitochondrial Ca^2+^ levels via regulation of MCUR1 and Ca^2+^ uniporter assembly [178].

Several MPT models have been proposed over the past 40 years. Indeed, only cyclophilin D is an integral component of the pore. In this case, proteins of the outer membrane and intermembrane space (VDAC, TSPO, HK, and CrK) are auxiliary in the assembly of the pore complex in intact mitochondria [179]. However, the question of which protein is the main pore component in the inner mitochondrial membrane has not yet been resolved. Until the mid-2000s the prevailing hypothesis was that such a channel-forming pore protein of the inner mitochondrial is adenine nucleotide translocator. This assumption was because the adenine nucleotide translocator inhibitors, atractyloside and carboxyatractyloside, stimulated pore opening, and bongkrekic acid showed an inhibitory effect. Adenine nucleotides carried by the translocator under normal conditions also suppressed the pore opening [175]. In addition, it was shown that ANT is able to bind to VDAC and hexokinase, as well as to cyclophilin D, forming channels in liposomes whose properties resemble MPT [180,181,182]. However, the discovery that the opening of the MPT pore also occurred in the mitochondria from ANT1 and ANT2 null mice led to the abandonment of this model [183]. After this, the idea of a phosphate carrier as a channel component of the MPT pore was considered [184].

According to the data of the last decade, mitochondrial ATP synthase is considered the main candidate for the role of the channel-forming component of the MPT pore, whose subunits and, in particular, OSCP, are able to combine with cyclophilin D, which, as expected, leads to pore opening [84,172,173,174]. In addition, it was shown that the OSCP subunit of ATP synthase contains a unique pH-sensitive histidine (H112), which has a significant modulating effect on the opening of the MPT pore [185]. Several suggestions have been made as to how ATP synthase can form an MPT pore channel. Two main hypotheses can be distinguished: (1) «dimer» and (2) «c-ring» [174]. According to the first one, ATP synthase dimers, but not monomers, conduct currents when inserted into planar lipid bilayers which are activated by Ca^2+^ and oxidizing agents and closed by ADP/Mg^2+^ (established MPT pore desensitizers) [186]. Further, it was shown that currents were strongly attenuated in yeast mutants that lacked ATP synthase subunits e and g necessary for the formation of dimers [187]. Indeed, channel formation by ATP synthase dimers was shown on mitochondria of evolutionarily distant species (*S. cerevisiae*, and *D. Melanogaster*), which makes this hypothesis quite convincing [187,188,189]. At the same time, it was found that the ATP synthase monomer is sufficient, and dimer formation is not required, for MPT pore activity [190]. According to an alternative «c-ring» hypothesis, the c-subunit of ATP synthase localized in the inner membrane of organelles may act as a channel component of the MPT pore [191,192]. In this case, it is assumed that Ca^2+^ and (or) ROS-induced dissociation of the F1 sector of ATP synthase leads to conformational changes in the c-ring of the Fo sector, which allows the formation of an MPT pore channel. However, this hypothesis runs into a series of contradictions. Indeed, in this case, the process of dissociation must be fast and reversible. First, there must be a quick detachment of the F1 sector, and second, the c-ring channel must be emptied and hydrated, which makes this hypothesis unlikely [174,193]. Moreover, the data of model experiments suggest that the hypothetical MPT pore based on the c-ring will have a significantly lower conductivity than that shown for MPT [194]. These data are supported by the results of patch-clamp measurements. Finally, it has recently been shown that mitochondria of mutant cells with disrupted c-subunits of ATP synthase still display a cyclosporin A-sensitive Ca^2+^-induced MPT pore opening and, moreover, in some cases demonstrate increased sensitivity to the induction of this process [195].

Recent data again bring us back to the question of ANT as a structural element of the MPT pore. Indeed, it has recently been shown that the MPT pore in c-subunit-deficient mitochondria is sensitive to cyclosporin A, ADP, and bongkrekic acid [195]. Finally, knockout of the genes of three ANT isoforms (not two, as in 2005) significantly increased mitochondrial resistance to MPT induction and the calcium capacity of organelles, nevertheless, did not prevent it [196]. These contradictions led to the emergence of a model for the joint participation of ANT and ATP-synthase in the MPT pore opening. It is assumed that, depending on the threshold concentration of Ca^2+^, these proteins will form channels of various currents that provide different modes of MPT functioning [197] and contribute to both the rapid release of Ca^2+^ and metabolites and the maintenance of the functioning of the futile cycles mentioned above.

It should also be mentioned that the search for MPT modulators made it possible to establish conditions when the mitochondrial Ca^2+^-dependent swelling was insensitive to cyclosporin A. This type of permeabilization includes the lipid pore induced by saturated fatty acids and Ca^2+^. In our previous papers, we described this type of permeabilization of the mitochondrial membrane in sufficient detail [84,198,199]. We found that the opening of this pore occurs by the mechanism of a chemotropic phase transition in a lipid bilayer. Indeed, palmitic acid in the presence of Ca^2+^ was able to permeabilize both natural and artificial membranes. The physiological significance of this pore has also been described [200]. The features of the formation and physiological significance of cyclosporin A-insensitive lipid pore are presented in more detail in our previous review [84].

### 3.3. The Sensitivity of Mitochondria of Various Tissues to the MPT Pore in Diabetes

The development of oxidative stress is known to accompany both diabetes and MPT pore induction. Based on this, one could suggest the direct involvement of MPT pore in the pathology of diabetes mellitus. However, a large number of studies on this issue do not give a clear answer. This seems to be due to the use of many diabetic models which renders a comparison of the results of different observers rather difficult. In addition, the sensitivity to the opening of the MPT pore in different organs and tissues during the development of diabetes mellitus varies greatly. Data on such differences are presented in Table 2 and in the subsequent part of this review.

#### 3.3.1. Pancreatic β-Cells

Cyclosporin A, an MPT pore inhibitor, has been shown to protect β-cells from glucotoxicity and prevent cell death [201]. In addition, substrate deprivation in INS-1 cells caused oxidative stress, followed by MPT pore opening and β-cell death [202]. Ablation of *Ppif*, the gene encoding cyclophilin D, normalized fasting glucose and glucose and insulin responses to an acute glucose challenge in adult mice maintained on a high-fat diet [203].

At the same time, the opening of the MPT pore may be an important process involved in the mechanism of insulin secretion in healthy pancreatic β-cells [221,222]. Cyclosporin A was shown to suppress glucose-induced insulin secretion in β-cells (lines MIN6 and INS-1) [221,222,223]. In this case, the authors noted the opening of the nonclassical MPT pore in β-cell mitochondria. The opening of such a pore leads to the conversion of electrical transmembrane potential into pH, thus mitochondrial respiration remains in a controlled state. At the same time, the pore opening was accompanied by a suppression of ROS production. On the other hand, it has been shown that the MPT pore may be responsible for non-glucose stimulated insulin secretion (NGSIS) in pancreatic islets. Non-esterified free fatty acids have been found to cause insulin secretion in pancreatic beta cells due to an increase in mitochondrial proton leak. Ablation of cyclophilin D or suppression of its activity by NIM811 significantly reduced both proton leak and NGSIS induced by NEFA [224]. However, other studies should be noted, where cyclosporin A did not inhibit insulin secretion in beta cells [225,226]. Thus, the MPT pore performs opposite functions in pancreatic cells, depending on physiological and pathological conditions.

#### 3.3.2. Heart

Numerous studies have shown that the heart mitochondria of diabetic rats are more sensitive to the induction of MPT pore than the mitochondria of control animals [159,227,228]. This increased mitochondrial sensitivity to pore opening and the development of oxidative stress is thought to underlie cardiomyopathy in diabetes mellitus. In the case of streptozotocin-induced type I diabetes the sensitivity to MPT does not change in the entire pool of heart mitochondria, but only in interfibrillar ones. This population of heart mitochondria in diabetic animals shows an increase in the amount of cyclophilin D, lower cytochrome c and Bcl-2 levels, and increased Bax levels. In this case endurance training reverted the hyperglycemia-induced CypD elevation and decreased mitochondrial Ca^2+^ release [209]. The sub-sarcolemmal population of heart mitochondria in *db/db* mice has been shown to undergo more severe pathological changes than the interfibrillar mitochondrial population [212].

It is noteworthy that in the heart of diabetic animals the level of protein molecules related to the MPT pore changed bi-directionally. This seems to be due to the different models of diabetes. Particularly, heart mitochondria show a significant increase in the level of ATP synthase and creatine kinase subunits in the model of streptozotocin-induced diabetes, which may reduce the resistance of organelles to MPT pore opening [215]. On the other hand, the level of ATP synthase was reduced in the hearts of *db/db* mice [212,229]. The hearts of rats (Zucker Fa/fa or streptozotocin-treated) show no change in the level of ANT1 [215,217,227], but demonstrate an increase in the number of open thiol groups in this protein, which, presumably, promotes ROS-induced pore induction associated with this protein [217,227]. In other studies, in the case of the streptozotocin model of diabetic rats and *db/db* mice, the heart showed a decrease in the level of ANT, which contradicts the decrease in mitochondrial resistance to MPT opening [212,230,231]. Analysis of the VDAC level in the cells of the cardiovascular system in diabetes mellitus also did not reveal significant patterns. It increased, decreased, or did not change [215,230,231]. In a few studies, it was also noted that diabetes mellitus increases the interaction of cyclophilin D with the hypothetical channel-forming components of the pore. In particular, the hearts of Goto-Kakizaki rats show an increase in the interaction of cyclophilin D with mitochondrial inorganic phosphate carrier, which sensitizes the MPT pore. In this case, a chemical chaperone, 4-phenylbutyric acid, attenuated the changes in CypD-PiC interaction and restored responses of calcium retention capacity and infarct size to erythropoietin in GK [228].

As noted above, the MPT pore and oxidative stress are thought to underlie diabetic cardiomyopathy. Therefore, the correction of this pathology with MPT pore inhibitors looks attractive. Indeed, it has been shown that administration of cyclosporin A, NIM811, MTP-131, or the mitochondrial calcium uniporter blocker minocycline at the onset of reperfusion, reduced infarct sizes in both control and diabetic hearts [222,232]. These findings suggest that augmented susceptibility to injury in the diabetic heart is mediated by redox-dependent shifts in the MPT pore opening. On the other hand, it was demonstrated that inhibition of the MPT pore with cyclosporin A failed to restore cardioprotection in the prediabetic but normoglycaemic heart of Zucker obese rats in vivo [233].

#### 3.3.3. Skeletal Muscles

Skeletal muscle mitochondria, like heart mitochondria, become more sensitive to the induction of the MPT pore during the development of diabetes mellitus (calcium retention capacity decreases, oxidative stress develops) [204,218]. Mice lacking CypD were protected from high fat diet-induced glucose intolerance due to increased glucose uptake in skeletal muscle. The mitochondria in *CypD* knockout muscle were resistant to diet-induced swelling and had improved calcium retention capacity compared to controls; however, no changes were observed in muscle oxidative damage, insulin signaling, lipotoxic lipid accumulation or mitochondrial bioenergetics. Cyclosporin A prevented insulin resistance and enhances glut4 expression in cell culture. It was suggested that the protective effect of CsA on insulin resistance is associated with inhibition of the pore, but not the effect on signaling pathways [218].

It was demonstrated that ANT1 protein abundance was not significantly reduced in skeletal muscle mitochondria of ZDF rats compared with the wild-type control rats [213]. At the same time, it was also shown that ANT1-deficient animals are insulin-hypersensitive, glucose-tolerant, and resistant to high fat diet-induced toxicity. In ANT1-deficient skeletal muscle, mitochondrial gene expression is induced in association with the hyperproliferation of mitochondria [234].

At the same time, mitochondria obtained from gastrocnemius muscles of streptozotocin-induced T1DM mice showed a decrease in mitochondrial respiratory control ratio and to decreased calcium- dependent MPT pore. [205]. Thus, the diabetic model (or its type) has a significant effect on the resistance of mitochondria to the induction of the MPT pore in skeletal muscle cells. More comprehensive research is required using various models to determine the more precise direction of these changes.

#### 3.3.4. Neural Tissue

The development of diabetes mellitus is also known to increase the sensitivity of brain mitochondria to MPT [206,210,235]. In patients with diabetes, brain CypD protein levels were increased. Diabetes triggers enhancement of F1F0 ATP synthase—CypD interaction, which in turn leads to MTP opening [210]. The sensitivity of brain mitochondria to MPT decreased in cyclophilin D-deficient mice with streptozotocin-induced diabetes. ATP synthesis deficits, oxidative stress and mitochondrial dysfunction were also suppressed in these mice [210].

Cyclosporin A also prevented seizure from occurring and virtually eliminated neuronal necrosis under hyperglycemia [219]. Moreover, the administration of cyclosporin A blocked the osmotic swelling of Müller cells in retinal slices from diabetic animals [236]. Hyperglycemia has been shown to be accompanied by significant rearrangements of the mitochondrial proteome and, especially, an increase in the level of ANT1 in the mitochondria of nerve cells in in vitro experiments [214]. However, as with skeletal muscle mitochondria, there is evidence that streptozotocin-induced diabetes did not promote brain mitochondrial dysfunction, suggesting that oxidative stress associated with type 1 diabetes is not directly related to mitochondrial dysfunction, but probably is related to extramitochondrial factor(s) [207].

#### 3.3.5. Kidney

As in the case of skeletal muscle and brain mitochondria, there are conflicting data on the role of the MPT pore in the development of mitochondrial dysfunction in diabetic kidney cells. On the one hand, kidney mitochondria from the diabetic animals had an increased susceptibility to the induction of the MPT pore by Ca^2+^ [208]. CypD loss triggers a metabolic shift in mouse kidneys towards glycolysis and Krebs cycle activity. The shift is accompanied by increased glucose consumption and a transcriptional upregulation of effectors of glucose metabolism in the kidney [237]. On the other hand, it was recently shown that cyclophilin D-null mice are not protected against T1DM-induced albuminuria. Alisporivir (a non-immunosuppressive CsA analog) did not improve renal function nor pathology in *db/db* mice. In these mice, alisporivir had no effect on changes in the structure and function of mitochondria. Thus, the authors concluded that direct targeting of cyclophilin D will likely not improve renal outcomes [211]. It should be noted that streptozotocin administration in C57BL/6 mice led to a decrease in the level of ATP synthase in kidney cells [211], while injection of streptozotocin in Sprague-Dawley rats caused an increase in the content of ATP synthase and its activity in renal mitochondria [216].

#### 3.3.6. Liver

Unlike other tissues, an increase in the resistance of liver mitochondria to MPT in type 1 and type 2 diabetes mellitus is described in most studies [41,114,238]. It was manifested as a Ca^2+^-dependent mitochondrial swelling delay and an increase in the Ca^2+^ capacity of organelles. However, an increase in the resistance of liver mitochondria to pore opening was manifested against the background of an increase in the level of lipid peroxidation products. It was suggested that the increased resistance of the organelles to MPT might be underlain by metabolic changes, such as an increase in the content of coenzyme Q or cardiolipin [114]. We recently demonstrated that streptozotocin-induced diabetes leads to a decrease in the levels of ANT1 and the c-subunit of ATP-synthase in liver mitochondria. This may be the cause of increased mitochondrial resistance to MPT in diabetes mellitus. It should be noted that an increase in the resistance of liver mitochondria to MPT pore opening is simultaneously accompanied by an increase in the sensitivity of organelles to cyclosporin A-insensitive lipid pore induced by palmitic acid and Ca^2+^ [41].

Cyclosporin A reduced the expression of PGC-1a in HepG2 cells. In addition, mtDNA level, mitochondria mass, ATP production, and cytochrome c oxidase activity were significantly reduced by treatment with cyclosporin A [220]. All this suggests that cyclosporin A inhibits mitochondrial biogenesis in liver cells. In addition, pharmacological and genetic inhibition of CypD has been shown to concomitantly reduce ER-mitochondria interactions and lead to hepatic insulin resistance and an increase in lipid accumulation [239].

However, it should be noted that the liver mitochondria of female NOD/Unib mice (non-obese diabetic mouse model) were more sensitive to MPT pore opening than the mitochondria of the control animals (Balb/c) [127].

In general, one could assume that in the case of pore opening, diabetes mellitus exhibits a pronounced tissue specificity (and, probably, model specificity). Diabetes mellitus enhances this process in some tissues, causing cardio- and myopathy and, ultimately, cell death. On the other hand, liver cells show adaptive changes that lead to a decrease in the sensitivity of mitochondria and cells to MPT. It seems reasonable to assume that this adaptation is necessary to maintain the primary functions of liver, including the neutralization of xenobiotics. The presence of enzyme systems for the xenobiotic neutralization allows liver cells to specifically adapt to stresses.

### 3.4. MPT Pore as a Target for Diabetes Management

The issue of MPT pore as a target in the treatment of diabetes mellitus is rather complicated. According to a wide range of studies, cyclosporin A and other MPT pore inhibitors contribute to the suppression of mitochondrial dysfunction and improve the quality of life of animals [217,219,232]. Administration of high-dose cyclosporin A has been demonstrated to induce remission of type 1 diabetes mellitus [240]. On the other hand, the same cyclosporin causes suppression of mitochondrial biogenesis in liver cells [220]. Mice with the deletion of cyclophilin D show the development of hyperglycemia, insulin resistance, and glucose intolerance, albeit resistant to diet-induced obesity [241]. However, it should be noted that the in vivo interpretation of the effects of cyclosporin A as an MPT inhibitor may not always be correct. Cyclosporin A is a well-known immune suppressor, and its effect can be associated not only with the suppression of MPT, but also with the effect on various signaling pathways in humans and animals.

Synthetic and natural antidiabetic compounds exhibit a bi-directional effect on MPT pore opening. Notably, metformin has been shown to inhibit MPT pore opening in mitochondria, enhances biogenesis, and prevents cell death [61,242,243]. However, there is an evidence that metformin stimulates MPT in rat liver mitochondria [244]. The thiazolidinedione class of antidiabetic agents also shows a similar stimulating effect on MPT pore [245]. Moreover, troglitazone enhanced MPT pore induction in the liver of diabetic Zucker (*fa/fa*) rats [246]. The natural polyphenolic compound luteolin, reduces mortality from coronary artery diseases, including diabetes [107]. It has been shown to inhibit MPT pore opening [247]. On the other hand, the plant alkaloid berberine, used in traditional Chinese medicine and possessing antidiabetic properties [248], causes an inhibition of mitochondrial respiration and a decrease on Ca^2+^ loading capacity through induction of the mitochondrial permeability transition [249]. All this suggests that it is necessary to carefully approach the issue of diabetes mellitus therapy through the modulation of MPT pore activity.

## 4. Conclusions

Mitochondrial dysfunction is now widely recognized as an important factor in the development of diabetes. Management of mitochondrial dysfunction can undoubtedly contribute to improving glucose metabolism and seems promising in the context of diabetes therapy. At the same time, it must be remembered that diabetes affects the functioning of mitochondria in various organs and tissues with different intensities. This is clearly seen especially in the MPT pore opening in mitochondria. The use of a wide range of animal models, regimens, and duration of exposure to diabetic stress often leads to rather contradictory results regarding mitochondrial functional changes in diabetes. It is clear that mitochondria play a key role in both compensatory processes and pathological changes under diabetic stress. In this regard, it is necessary to carefully approach the issue of regulation of mitochondrial dysfunction in diabetes mellitus and conduct detailed comprehensive all organ studies of compounds that affect the functioning of mitochondria.

## Figures and Tables

**Table 1:** The mechanisms of diabetes-induced mitochondrial dysfunction in vital organs and tissues of human and animals.

	Pancreatic Islets	Heart	Skeletal Muscles	Brain	Kidney	Adipose	Liver
Biogenesis	↓ [11] ↑ [12]	↓↑ [13]	↓ [14]	↓ [15]	↓ [16]	↓ [17]	↓ [18] ↑ [19]
Mitophagy	↓ [20] ↑ [21]	↓ [22]	↓ [23]	↓ [24]	↓ [25] ↑ [26]	↑ [27]	↓ [28]
Fission/Fusion	↑/↓ [29]	↑/↓ [30]	↑ [31] ↓ [32]	↑/↓ [33]	↑/↓ [34]	↑ [35] ↓?	↑/↓ [36]
OXPHOS	↑ or ↓ or not changed, see review [37]						
ROS production	↑ [20]	↑ [38]	↑ [39]	↑ [40]	↑ [34]	↑ [17]	↑ [41]

**Table 2:** Tissue-specific susceptibility of mitochondria to MPT pore opening in diabetes mellitus models.

		Pancreatic β-Cells	Heart	Skeletal Muscles	Brain	Kidney	Liver
MPT resistance		↓ [201]	↓ [159]	↓ [204] ↑ [205]	↓ [206] ↑ [207]	↓ [208]	↑ [114]
MPT proteins							
	cyclophilin D	n.e.	↑ [209]	n.e.	↑ [210]	− [211]	− [41]
	ANT	n.e.	↓ [212]	− [213]	↑ [214]	n.e.	↓ [41]
	F0F1-ATPase	n.e.	↓ [212] ↑ [215]	n.e	n.e.	↓ [211] ↑ [216]	↓ [41]
MPT pore inhibitors		+ [201]	+ [217]	+ [218]	+ [219]	no effect [211]	− [220]

An increase or decrease in susceptibility to MPT, the level of mitochondrial proteins is indicated by arrows (↑ or ↓). The positive and negative effects of MPT pore inhibition on the functional state of the tissue are indicated as + and −, respectively. n.e.—no evidence (in literature).

## Abbreviations

ANT	Adenine nucleotide translocator
DRP1	Dynamin-related protein 1
GK	Goto-Kakizaki (GK) rats
MAM	Mitochondria-associated ER membranes
MCU	Mitochondrial Ca^2+^ uniporter
MICU	Mitochondrial Ca^2+^ uptake protein
MPT	Mitochondrial permeability transition
NCLX	Na^+^/Li^+^/Ca^2+^ exchanger
Parkin	E3 ubiquitin ligase Parkin
PGC-1α	Peroxisome proliferator-activated receptor γ (PPARγ) coactivator-1α
PINK1	PTEN-induced putative kinase 1
ROS	Reactive oxygen species
SGLT2	Sodium-glucose linked transporter 2
T1DM	Type 1 diabetes mellitus
T2DM	Type 2 diabetes mellitus
UCP	Uncoupling protein
VDAC	Voltage-dependent anion channel
ZDF	Zucker diabetic fatty (ZDF-Lepr fa/fa or fa/fa) rats

## References

[1] International Diabetes Federation (2019). IDF Diabetes Atlas.

[2] World Health Organization (2016). Global Report on Diabetes.

[3] Cristelo C., Azevedo C., Marques J.M., Nunes R., Sarmento B. (2020). SARS-CoV-2 and diabetes: New challenges for the disease. Diabetes Res. Clin. Pract..

[4] American Diabetes Association (2020). 2. Classification and diagnosis of diabetes: Standards of Medical Care in Diabetes—2020. Diabetes Care.

[5] Feldman E.L., Callaghan B.C., Pop-Busui R., Zochodne D.W., Wright D.E., Bennett D.L., Bril V., Russell J.W., Viswanathan V. (2019). Diabetic neuropathy. Nat. Rev. Dis. Primers.

[6] Miller R.G., Costacou T. (2019). Glucose management and the sex difference in excess cardiovascular disease risk in long-duration type 1 diabetes. Curr. Diab. Rep..

[7] Groop L.C., Eriksson J.G. (1992). The etiology and pathogenesis of non-insulin-dependent diabetes. Ann. Med..

[8] Montgomery M.K., Turner N. (2015). Mitochondrial dysfunction and insulin resistance: An update. Endocr. Connect..

[9] Prasun P. (2020). Mitochondrial dysfunction in metabolic syndrome. Biochim. Biophys. Acta Mol. Basis Dis..

[10] Yamada T., Ida T., Yamaoka Y., Ozawa K., Takasan H., Honjo I. (1975). Two distinct patterns of glucose intolerance in icteric rats and rabbits. Relationship to impaired liver mitochondria function. J. Lab. Clin. Med..

[11] Ling C., Del Guerra S., Lupi R., Ronn T., Granhall C., Luthman H., Masiello P., Marchetti P., Groop L., Del Prato S. (2008). Epigenetic regulation of PPARGC1A in human type 2 diabetic islets and effect on insulin secretion. Diabetologia.

[12] Yoon J.C., Xu G., Deeney J.T., Yang S.N., Rhee J., Puigserver P., Levens A.R., Yang R., Zhang C.Y., Lowell B.B. (2003). Suppression of beta cell energy metabolism and insulin release by PGC-1alpha. Dev. Cell..

[13] Gollmer J., Zirlik A., Bugger H. (2020). Mitochondrial Mechanisms in Diabetic Cardiomyopathy. Diabetes Metab. J..

[14] Patti M.E., Butte A.J., Crunkhorn S., Cusi K., Berria R., Kashyap S., Miyazaki Y., Kohane I., Costello M., Saccone R. (2003). Coordinated reduction of genes of oxidative metabolism in humans with insulin resistance and diabetes: Potential role of PGC1 and NRF1. Proc. Natl. Acad. Sci. USA.

[15] Choi J., Chandrasekaran K., Inoue T., Muragundla A., Russell J.W. (2014). PGC-1α regulation of mitochondrial degeneration in experimental diabetic neuropathy. Neurobiol. Dis..

[16] Guo K., Lu J., Huang Y., Wu M., Zhang L., Yu H., Zhang M., Bao Y., He J.C., Chen H. (2015). Protective role of PGC-1α in diabetic nephropathy is associated with the inhibition of ROS through mitochondrial dynamic remodeling. PLoS ONE.

[17] Gao C.L., Zhu C., Zhao Y.P., Chen X.H., Ji C.B., Zhang C.M., Zhu J.G., Xia Z.K., Tong M.L., Guo X.R. (2010). Mitochondrial dysfunction is induced by high levels of glucose and free fatty acids in 3T3-L1 adipocytes. Mol. Cell Endocrinol..

[18] Holmstrom M.H., Iglesias-Gutierrez E., Zierath J.R., Garcia-Roves P.M. (2012). Tissue-specific control of mitochondrial respiration in obesity-related insulin resistance and diabetes. Am. J. Physiol. Endocrinol. Metab..

[19] Yoon J.C., Puigserver P., Chen G., Donovan J., Wu Z., Rhee J., Adelmant G., Stafford J., Kahn C.R., Granner D.K. (2001). Control of hepatic gluconeogenesis through the transcriptional coactivator PGC-1. Nature.

[20] Bhansali S., Bhansali A., Walia R., Saikia U.N., Dhawan V. (2017). Alterations in mitochondrial oxidative stress and mitophagy in subjects with prediabetes and type 2 diabetes mellitus. Front. Endocrinol..

[21] Ebato C., Uchida T., Arakawa M., Komatsu M., Ueno T., Komiya K., Azuma K., Hirose T., Tanaka K., Kominami E. (2008). Autophagy is important in islet homeostasis and compensatory increase of beta cell mass in response to high-fat diet. Cell Metab..

[22] Yu W., Gao B., Li N., Wang J., Qiu C., Zhang G., Liu M., Zhang R., Li C., Ji G. (2017). Sirt3 deficiency exacerbates diabetic cardiac dysfunction: Role of Foxo3A-Parkin-mediated mitophagy. Biochim. Biophys. Acta Mol. Basis Dis..

[23] Gundersen A.E., Kugler B.A., McDonald P.M., Veraksa A., Houmard J.A., Zou K. (2020). Altered mitochondrial network morphology and regulatory proteins in mitochondrial quality control in myotubes from severely obese humans with or without type 2 diabetes. Appl. Physiol. Nutr. Metab..

[24] Santos R.X., Correia S.C., Alves M.G., Oliveira P.F., Cardoso S., Carvalho C., Seiça R., Santos M.S., Moreira P.I. (2014). Mitochondrial quality control systems sustain brain mitochondrial bioenergetics in early stages of type 2 diabetes. Mol. Cell Biochem..

[25] Zhao Y., Sun M. (2020). Metformin rescues Parkin protein expression and mitophagy in high glucose-challenged human renal epithelial cells by inhibiting NF-κB via PP2A activation. Life Sci..

[26] Jiang X.S., Chen X.M., Hua W., He J.L., Liu T., Li X.J., Wan J.M., Gan H., Du X.G. (2020). PINK1/Parkin mediated mitophagy ameliorates palmitic acid-induced apoptosis through reducing mitochondrial ROS production in podocytes. Biochem. Biophys. Res. Commun..

[27] Kosacka J., Kern M., Klöting N., Paeschke S., Rudich A., Haim Y., Gericke M., Serke H., Stumvoll M., Bechmann I. (2015). Autophagy in adipose tissue of patients with obesity and type 2 diabetes. Mol. Cell Endocrinol..

[28] Liu P., Lin H., Xu Y., Zhou F., Wang J., Liu J., Zhu X., Guo X., Tang Y., Yao P. (2018). Frataxin-mediated PINK1-Parkin-dependent mitophagy in hepatic steatosis: The protective effects of quercetin. Mol. Nutr. Food Res..

[29] Jezek P., Dlaskova A. (2019). Dynamic of mitochondrial network, cristae, and mitochondrial nucleoids in pancreatic β-cells. Mitochondrion.

[30] Hu L., Ding M., Tang D., Gao E., Li C., Wang K., Qi B., Qiu J., Zhao H., Chang P. (2019). Targeting mitochondrial dynamics by regulating Mfn2 for therapeutic intervention in diabetic cardiomyopathy. Theranostics.

[31] Fealy C.E., Mulya A., Lai N., Kirwan J.P. (2014). Exercise training decreases activation of the mitochondrial fission protein dynamin-related protein-1 in insulin-resistant human skeletal muscle. J. Appl. Physiol..

[32] Peyravi A., Yazdanpanahi N., Nayeri H., Hosseini S.A. (2020). The effect of endurance training with crocin consumption on the levels of MFN2 and DRP1 gene expression and glucose and insulin indices in the muscle tissue of diabetic rats. J. Food Biochem..

[33] Chen L., Huang J., Li X.C., Liu S.Y., Li Y.H., Wang Q., Yang J.J., Cao H.M., Hu Q.K., He L.J. (2019). High-glucose induced mitochondrial dynamics disorder of spinal cord neurons in diabetic rats and its effect on mitochondrial spatial distribution. Spine.

[34] Lee W.C., Chau Y.Y., Ng H.Y., Chen C.H., Wang P.W., Liou C.W., Lin T.K., Chen J.B. (2019). Empagliflozin protects HK-2 cells from high glucose-mediated injuries via a mitochondrial mechanism. Cells.

[35] Lahera V., de Las Heras N., Lopez-Farre A., Manucha W., Ferder L. (2017). Role of mitochondrial dysfunction in hypertension and obesity. Curr. Hypertens. Rep..

[36] Xu Z., Zhang L., Li X., Jiang Z., Sun L., Zhao G., Zhou G., Zhang H., Shang J., Wang T. (2015). Mitochondrial fusion/fission process involved in the improvement of catalpol on high glucose-induced hepatic mitochondrial dysfunction. Acta Biochim. Biophys. Sin..

[37] Lewis M.T., Kasper J.D., Bazil J.N., Frisbee J.C., Wiseman R.W. (2019). Quantification of mitochondrial oxidative phosphorylation in metabolic disease: Application to type 2 diabetes. Int. J. Mol. Sci..

[38] Ni R., Cao T., Xiong S., Ma J., Fan G.C., Lacefield J.C., Lu Y., Le Tissier S., Peng T. (2016). Therapeutic inhibition of mitochondrial reactive oxygen species with mito-TEMPO reduces diabetic cardiomyopathy. Free Radic. Biol. Med..

[39] Szkudelska K., Okulicz M., Hertig I., Szkudelski T. (2020). Resveratrol ameliorates inflammatory and oxidative stress in type 2 diabetic Goto-Kakizaki rats. Biomed. Pharmacother..

[40] Raza H., John A., Howarth F.C. (2015). Increased oxidative stress and mitochondrial dysfunction in zucker diabetic rat liver and brain. Cell. Physiol. Biochem..

[41] Belosludtsev K.N., Talanov E.Y., Starinets V.S., Agafonov A.V., Dubinin M.V., Belosludtseva N.V. (2019). Transport of Ca^2+^ and Ca^2+^—Dependent permeability transition in rat liver mitochondria under the streptozotocin-induced type I diabetes. Cells.

[42] Joost H.-G., Al-Hasani H., Schürmann A. (2012). Animal Models in Diabetes Research.

[43] Gvazava I.G., Rogovaya O.S., Borisov M.A., Vorotelyak E.A., Vasiliev A.V. (2018). Pathogenesis of type 1 diabetes mellitus and rodent experimental models. Acta Nat..

[44] King A.J. (2012). The use of animal models in diabetes research. Br. J. Pharmacol..

[45] Rius-Pérez S., Torres-Cuevas I., Millán I., Ortega A.L., Pérez S. (2020). PGC-1α, Inflammation, and oxidative stress: An integrative view in metabolism. Oxid. Med. Cell Longev..

[46] Zhu L., Wang Q., Zhang L., Fang Z., Zhao F., Lv Z., Gu Z., Zhang J., Wang J., Zen K. (2010). Hypoxia induces PGC-1α expression and mitochondrial biogenesis in the myocardium of TOF patients. Cell Res..

[47] Herzig S., Long F., Jhala U.S., Hedrick S., Quinn R., Bauer A., Rudolph D., Schutz G., Yoon C., Puigserver P. (2001). CREB regulates hepatic gluconeogenesis through the coactivator PGC-1. Nature.

[48] Schaeffer P.J., Wende A.R., Magee C.J., Neilson J.R., Leone T.C., Chen F., Kelly D.P. (2004). Calcineurin and calcium/calmodulin-dependent protein kinase activate distinct metabolic gene regulatory programs in cardiac muscle. J. Biol. Chem..

[49] Nisoli E., Clementi E., Paolucci C., Cozzi V., Tonello C., Sciorati C., Bracale R., Valerio A., Francolini M., Moncada S. (2003). Mitochondrial biogenesis in mammals: The role of endogenous nitric oxide. Science.

[50] Handschin C., Rhee J., Lin J., Tarr P.T., Spiegelman B.M. (2003). An autoregulatory loop controls peroxisome proliferator-activated receptor gamma coactivator 1alpha expression in muscle. Proc. Natl. Acad. Sci. USA.

[51] Gureev A.P., Shaforostova E.A., Popov V.N. (2019). Regulation of mitochondrial biogenesis as a way for active longevity: Interaction between the Nrf2 and PGC-1α signaling pathways. Front. Genet..

[52] Di W., Lv J., Jiang S., Lu C., Yang Z., Ma Z., Hu W., Yang Y., Xu B. (2018). PGC-1: The energetic regulator in cardiac metabolism. Curr. Issues Mol. Biol..

[53] Pinti M.V., Fink G.K., Hathaway Q.A., Durr A.J., Kunovac A., Hollander J.M. (2019). Mitochondrial dysfunction in type 2 diabetes mellitus: An organ-based analysis. Am. J. Physiol. Endocrinol. Metab..

[54] Makrecka-Kuka M., Liepinsh E., Murray A.J., Lemieux H., Dambrova M., Tepp K., Puurand M., Kaambre T., Han W.H., de Goede P. (2020). Altered mitochondrial metabolism in the insulin-resistant heart. Acta Physiol..

[55] Wu H., Deng X., Shi Y., Su Y., Wei J., Duan H. (2016). PGC-1α, glucose metabolism and type 2 diabetes mellitus. J. Endocrinol..

[56] Stump C.S., Short K.R., Bigelow M.L., Schimke J.M., Nair K.S. (2003). Effect of insulin on human skeletal muscle mitochondrial ATP production, protein synthesis, and mRNA transcripts. Proc. Natl. Acad. Sci. USA.

[57] Karakelides H., Asmann Y.W., Bigelow M.L., Short K.R., Dhatariya K., Coenen-Schimke J., Kahl J., Mukhopadhyay D., Nair K.S. (2007). Effect of insulin deprivation on muscle mitochondrial ATP production and gene transcript levels in type 1 diabetic subjects. Diabetes.

[58] Toledo F.G., Menshikova E.V., Ritov V.B., Azuma K., Radikova Z., DeLany J., Kelley D.E. (2007). Effects of physical activity and weight loss on skeletal muscle mitochondria and relationship with glucose control in type 2 diabetes. Diabetes.

[59] Fujisawa K., Nishikawa T., Kukidome D., Imoto K., Yamashiro T., Motoshima H., Matsumura T., Araki E. (2009). TZDs reduce mitochondrial ROS production and enhance mitochondrial biogenesis. Biochem. Biophys. Res. Commun..

[60] Shao Q., Meng L., Lee S., Tse G., Gong M., Zhang Z., Zhao J., Zhao Y., Li G., Liu T. (2019). Empagliflozin, a sodium glucose co-transporter-2 inhibitor, alleviates atrial remodeling and improves mitochondrial function in high-fat diet/streptozotocin-induced diabetic rats. Cardiovasc. Diabetol..

[61] Docrat T.F., Nagiah S., Naicker N., Baijnath S., Singh S., Chuturgoon A.A. (2020). The protective effect of metformin on mitochondrial dysfunction and endoplasmic reticulum stress in diabetic mice brain. Eur. J. Pharmacol..

[62] Mootha V.K., Lindgren C.M., Eriksson K.F., Subramanian A., Sihag S., Lehar J., Puigserver P., Carlsson E., Ridderstråle M., Laurila E. (2003). PGC-1alpha-responsive genes involved in oxidative phosphorylation are coordinately downregulated in human diabetes. Nat. Genet..

[63] Ploumi C., Daskalaki I., Tavernarakis N. (2017). Mitochondrial biogenesis and clearance: A balancing act. FEBS J..

[64] Koo S.H., Satoh H., Herzig S., Lee C.H., Hedrick S., Kulkarni R., Evans R.M., Olefsky J., Montminy M. (2004). PGC-1 promotes insulin resistance in liver through PPAR-alpha-dependent induction of TRB-3. Nat. Med..

[65] Masini M., Martino L., Marselli L., Bugliani M., Boggi U., Filipponi F., Marchetti P., De Tata V. (2017). Ultrastructural alterations of pancreatic beta cells in human diabetes mellitus. Diabetes Metab. Res. Rev..

[66] Boudina S., Abel E.D. (2006). Mitochondrial uncoupling: A key contributor to reduced cardiac efficiency in diabetes. Physiology.

[67] Shen X., Zheng S., Thongboonkerd V., Xu M., Pierce W.M., Klein J.B., Epstein P.N. (2004). Cardiac mitochondrial damage and biogenesis in a chronic model of type 1 diabetes. Am. J. Physiol. Endocrinol. Metab..

[68] Yang X., Pan W., Xu G., Chen L. (2020). Mitophagy: A crucial modulator in the pathogenesis of chronic diseases. Clin. Chim. Acta.

[69] Bakula D., Scheibye-Knudsen M. (2020). MitophAging: Mitophagy in aging and disease. Front. Cell Dev. Biol..

[70] Watada H., Fujitani Y. (2015). Minireview: Autophagy in pancreatic β-cells and its implication in diabetes. Mol. Endocrinol..

[71] Rocha M., Apostolova N., Diaz-Rua R., Muntane J., Victor V.M. (2020). Mitochondria and T2D: Role of autophagy, ER stress, and inflammasome. Trends Endocrinol. Metab..

[72] Wang Y., Liang B., Lau W.B., Du Y., Guo R., Yan Z., Gan L., Yan W., Zhao J., Gao E. (2017). Restoring diabetes-induced autophagic flux arrest in ischemic/reperfused heart by ADIPOR (adiponectin receptor) activation involves both AMPK-dependent and AMPK-independent signaling. Autophagy.

[73] Tao A., Xu X., Kvietys P., Kao R., Martin C., Rui T. (2018). Experimental diabetes mellitus exacerbates ischemia/reperfusion-induced myocardial injury by promoting mitochondrial fission: Role of down-regulation of myocardial Sirt1 and subsequent Akt/Drp1 interaction. Int. J. Biochem. Cell Biol..

[74] Packer M. (2020). Autophagy-dependent and -independent modulation of oxidative and organellar stress in the diabetic heart by glucose-lowering drugs. Cardiovasc. Diabetol..

[75] Hoshino A., Ariyoshi M., Okawa Y., Kaimoto S., Uchihashi M., Fukai K., Iwai-Kanai E., Ikeda K., Ueyama T., Ogata T. (2014). Inhibition of p53 preserves Parkin-mediated mitophagy and pancreatic β-cell function in diabetes. Proc. Natl. Acad. Sci. USA.

[76] Bhansali S., Bhansali A., Dutta P., Walia R., Dhawan V. (2020). Metformin upregulates mitophagy in patients with T2DM: A randomized placebo-controlled study. J. Cell Mol. Med..

[77] Lee Y.H., Kim S.H., Kang J.M., Heo J.H., Kim D.J., Park S.H., Sung M., Kim J., Oh J., Yang D.H. (2019). Empagliflozin attenuates diabetic tubulopathy by improving mitochondrial fragmentation and autophagy. Am. J. Physiol. Renal. Physiol..

[78] Hawley S.A., Ford R.J., Smith B.K., Gowans G.J., Mancini S.J., Pitt R.D., Day E.A., Salt I.P., Steinberg G.R., Hardie D.G. (2016). The Na^+^/glucose cotransporter inhibitor canagliflozin activates AMPK by inhibiting mitochondrial function and increasing cellular AMP levels. Diabetes.

[79] Rovira-Llopis S., Banuls C., Diaz-Morales N., Hernandez-Mijares A., Rocha M., Victor V.M. (2017). Mitochondrial dynamics in type 2 diabetes: Pathophysiological implications. Redox Biol..

[80] Buhlman L., Damiano M., Bertolin G., Ferrando-Miguel R., Lombès A., Brice A., Corti O. (2014). Functional interplay between Parkin and Drp1 in mitochondrial fission and clearance. Biochim. Biophys. Acta.

[81] Chan D.C. (2020). Mitochondrial dynamics and its involvement in disease. Annu. Rev. Pathol..

[82] Loson O.C., Song Z., Chen H., Chan D.C. (2013). Fis1, Mff, MiD49, and MiD51 mediate Drp1 recruitment in mitochondrial fission. Mol. Biol. Cell.

[83] De Brito O.M., Scorrano L. (2008). Mitofusin 2 tethers endoplasmic reticulum to mitochondria. Nature.

[84] Belosludtsev K.N., Dubinin M.V., Belosludtseva N.V., Mironova G.D. (2019). Mitochondrial Ca^2+^ transport: Mechanisms, molecular structures, and role in cells. Biochemistry.

[85] Sabouny R., Shutt T.E. (2020). Reciprocal regulation of mitochondrial fission and fusion. Trends Biochem. Sci..

[86] Frezza C., Cipolat S., de Brito O., Micaroni M., Beznoussenko G.V., Rudka T., Bartoli D., Polishuck R.S., Danial N.N., De Strooper B. (2006). OPA1 controls apoptotic cristae remodeling independently from mitochondrial fusion. Cell.

[87] Liu R., Jin P., Yu L., Wang Y., Han L., Shi T., Li X. (2014). Impaired mitochondrial dynamics and bioenergetics in diabetic skeletal muscle. PLoS ONE.

[88] Makino A., Scottm B.T., Dillmann W.H. (2010). Mitochondrial fragmentation and superoxide anion production in coronary endothelial cells from a mouse model of type 1 diabetes. Diabetologia.

[89] Yu J., Maimaitili Y., Xie P., Wu J.J., Wang J., Yang Y.N., Ma H.P., Zheng H. (2017). High glucose concentration abrogates sevoflurane post-conditioning cardioprotection by advancing mitochondrial fission but dynamin-related protein 1 inhibitor restores these effects. Acta Physiol..

[90] Ding M., Liu C., Shi R., Yu M., Zeng K., Kang J., Fu F., Mi M. (2020). Mitochondrial fusion promoter restores mitochondrial dynamics balance and ameliorates diabetic cardiomyopathy in an optic atrophy 1-dependent way. Acta Physiol..

[91] Peng L., Men X., Zhang W., Wang H., Xu S., Fang Q., Liu H., Yang W., Lou J. (2012). Involvement of dynamin-related protein 1 in free fatty acid-induced INS-1-derived cell apoptosis. PLoS ONE.

[92] Jheng H.F., Tsai P.J., Guo S.M., Kuo L.H., Chang C.S., Su I.J., Chang C.R., Tsai Y.S. (2012). Mitochondrial fission contributes to mitochondrial dysfunction and insulin resistance in skeletal muscle. Mol. Cell. Biol..

[93] Burman J.L., Pickles S., Wang C., Sekine S., Vargas J., Zhang Z., Youle A.M., Nezich C.L., Wu X., Hammer J.A. (2017). Mitochondrial fission facilitates the selective mitophagy of protein aggregates. J. Cell Biol..

[94] Zhou H., Wang S., Zhu P., Hu S., Chen Y., Ren J. (2018). Empagliflozin rescues diabetic myocardial microvascular injury via AMPK-mediated inhibition of mitochondrial fission. Redox Biol..

[95] Durak A., Olgar Y., Degirmenci S., Akkus E., Tuncay E., Turan B. (2018). A SGLT2 inhibitor dapagliflozin suppresses prolonged ventricular-repolarization through augmentation of mitochondrial function in insulin-resistant metabolic syndrome rats. Cardiovasc. Diabetol..

[96] Li A., Zhang S., Li J., Liu K., Huang F., Liu B. (2016). Metformin and resveratrol inhibit Drp1-mediated mitochondrial fission and prevent ER stress-associated NLRP3 inflammasome activation in the adipose tissue of diabetic mice. Mol. Cell. Endocrinol..

[97] Liu H., Xiang H., Zhao S., Sang H., Lv F., Chen R., Shu Z., Chen A.F., Chen S., Lu H. (2019). Vildagliptin improves high glucose-induced endothelial mitochondrial dysfunction via inhibiting mitochondrial fission. J. Cell. Mol. Med..

[98] Magnusson I., Rothman D.L., Katz L.D., Shulman R.G., Shulman G.I. (1992). Increased rate of gluconeogenesis in type II diabetes mellitus. A 13C nuclear magnetic resonance study. J. Clin. Investig..

[99] Petersen K.F., Price T.B., Bergeron R. (2004). Regulation of net hepatic glycogenolysis and gluconeogenesis during exercise: Impact of type 1 diabetes. J. Clin. Endocrinol. Metab..

[100] Barger P.M., Kelly D.P. (2000). PPAR signaling in the control of cardiac energy metabolism. Trends Cardiovasc. Med..

[101] Ruderman N.B., Dean D. (1998). Malonyl CoA, long chain fatty acyl CoA and insulin resistance in skeletal muscle. J. Basic Clin. Physiol. Pharmacol..

[102] Adams S.H. (2011). Emerging perspectives on essential amino acid metabolism in obesity and the insulin-resistant state. Adv. Nutr..

[103] Yin J., Ren W., Chen S., Li Y., Han H., Gao J., Liu G., Wu X., Li T., Woo Kim S. (2018). Metabolic regulation of methionine restriction in diabetes. Mol. Nutr. Food Res..

[104] Mogensen M., Sahlin K., Fernström M., Glintborg D., Vind B.F., Beck-Nielsen H., Højlund K. (2007). Mitochondrial respiration is decreased in skeletal muscle of patients with type 2 diabetes. Diabetes.

[105] Koentges C., Konig A., Pfeil K., Holscher M.E., Schnick T., Wende A.R., Schrepper A., Cimolai M.C., Kersting S., Hoffmann M.M. (2015). Myocardial mitochondrial dysfunction in mice lacking adiponectin receptor 1. Basic Res. Cardiol..

[106] Bombicino S.S., Iglesias D.E., Mikusic I., D’Annunzio V., Gelpi R.J., Boveris A., Valdez L.B. (2016). Diabetes impairs heart mitochondrial function without changes in resting cardiac performance. Int. J. Biochem. Cell Biol..

[107] Cortés-Rojo C., Vargas-Vargas M.A., Olmos-Orizaba B.E., Rodríguez-Orozco A.R., Calderón-Cortés E. (2020). Interplay between NADH oxidation by complex I, glutathione redox state and sirtuin-3, and its role in the development of insulin resistance. Biochim. Biophys. Acta Mol. Basis Dis..

[108] Ni R., Zheng D., Xiong S., Hill D.J., Sun T., Gardiner R.B., Fan G.C., Lu Y., Abel E.D., Greer P.A. (2016). Mitochondrial calpain-1 disrupts ATP synthase and induces superoxide generation in type 1 diabetic hearts: A novel mechanism contributing to diabetic cardiomyopathy. Diabetes.

[109] Ong S.B., Lee W.H., Shao N.Y., Ismail N.I., Katwadi K., Lim M.M., Kwek X.Y., Michel N.A., Li J., Newson J. (2019). Calpain inhibition restores autophagy and prevents mitochondrial fragmentation in a human iPSC model of diabetic endotheliopathy. Stem. Cell Reports..

[110] Antoun G., McMurray F., Thrush A.B., Patten D.A., Peixoto A.C., Slack R.S., McPherson R., Dent R., Harper M.E. (2015). Impaired mitochondrial oxidative phosphorylation and supercomplex assembly in rectus abdominis muscle of diabetic obese individuals. Diabetologia.

[111] Solsona-Vilarrasa E., Fucho R., Torres S., Nuñez S., Nuño-Lámbarri N., Enrich C., García-Ruiz C., Fernández-Checa J.C. (2019). Cholesterol enrichment in liver mitochondria impairs oxidative phosphorylation and disrupts the assembly of respiratory supercomplexes. Redox Biol..

[112] Starinets V.S., Lebedeva E.V., Mikheeva I.B., Belosludtseva N.V., Dubinin M.V., Belosludtsev K.N. (2019). Ultrastructural and functional changes in liver mitochondria in a rat model of type I diabetes mellitus. Biophysics.

[113] Ferreira F.M., Palmeira C.M., Seiça R., Santos M.S. (1999). Alterations of liver mitochondrial bioenergetics in diabetic Goto-Kakizaki rats. Metabolism.

[114] Ferreira F.M., Seiça R., Oliveira P.J., Coxito P.M., Moreno A.J., Palmeira C.M., Santos M.S. (2003). Diabetes induces metabolic adaptations in rat liver mitochondria: Role of coenzyme Q and cardiolipin contents. Biochim. Biophys. Acta.

[115] Bridges H.R., Jones A.J., Pollak M.N., Hirst J. (2014). Effects of metformin and other biguanides on oxidative phosphorylation in mitochondria. Biochem. J..

[116] Martín-Rodríguez S., de Pablos-Velasco P., Calbet J.A.L. (2020). Mitochondrial complex I inhibition by metformin: Drug-exercise interactions. Trends Endocrinol. Metab..

[117] García-Ruiz I., Solís-Muñoz P., Fernández-Moreira D., Muñoz-Yagüe T., Solís-Herruzo J.A. (2013). Pioglitazone leads to an inactivation and disassembly of complex I of the mitochondrial respiratory chain. BMC Biol..

[118] Seydi E., Servati T., Samiei F., Naserzadeh P., Pourahmad J. (2020). Toxicity of pioglitazone on mitochondria isolated from brain and heart: An analysis for probable drug-induced neurotoxicity and cardiotoxicity. Drug Res..

[119] Fedorenko A., Lishko P.V., Kirichok Y. (2012). Mechanism of fatty-acid-dependent UCP1 uncoupling in brown fat mitochondria. Cell.

[120] Samartsev V.N. (2000). Fatty acids as uncouplers of oxidative phosphorylation. Biochemistry.

[121] Hidaka S., Kakuma T., Yoshimatsu H., Sakino H., Fukuchi S., Sakata T. (1999). Streptozotocin treatment upregulates uncoupling protein 3 expression in the rat heart. Diabetes.

[122] He Y., Luan Z., Fu X., Xu X. (2016). Overexpression of uncoupling protein 2 inhibits the high glucose-induced apoptosis of human umbilical vein endothelial cells. Int. J. Mol. Med..

[123] Kim J.D., Yoon N.A., Jin S., Diano S. (2019). Microglial UCP2 mediates inflammation and obesity induced by high-fat feeding. Cell Metab..

[124] Boudina S., Han Y.H., Pei S., Tidwell T.J., Henrie B., Tuinei J., Olsen C., Sena S., Abel E.D. (2012). UCP3 regulates cardiac efficiency and mitochondrial coupling in high fat-fed mice but not in leptin-deficient mice. Diabetes.

[125] Yaribeygi H., Sathyapalan T., Atkin S.L., Sahebkar A. (2020). Molecular mechanisms linking oxidative stress and diabetes mellitus. Oxid. Med. Cell Longev..

[126] Fisher-Wellman K.H., Mattox T.A., Thayne K., Katunga L.A., La Favor J.D., Neufer P.D., Hickner R.C., Wingard C.J., Anderson E.J. (2013). Novel role for thioredoxin reductase-2 in mitochondrial redox adaptations to obesogenic diet and exercise in heart and skeletal muscle. J. Physiol..

[127] Malaguti C., La Guardia P.G., Leite A.C., Oliveira D.N., de Lima Zollner R.L., Catharino R.R., Vercesi A.E., Oliveira H.C. (2014). Oxidative stress and susceptibility to mitochondrial permeability transition precedes the onset of diabetes in autoimmune non-obese diabetic mice. Free Radic. Res..

[128] Andreyev A.Y., Kushnareva Y.E., Murphy A.N., Starkov A.A. (2015). Mitochondrial ROS metabolism: 10 years later. Biochemistry.

[129] Wong H.S., Dighe P.A., Mezera V., Monternier P.A., Brand M.D. (2017). Production of superoxide and hydrogen peroxide from specific mitochondrial sites under different bioenergetic conditions. J. Biol. Chem..

[130] Kitada M., Ogura Y., Monno I., Koya D. (2019). Sirtuins and type 2 diabetes: Role in inflammation, oxidative stress, and mitochondrial function. Front. Endocrinol..

[131] Jing E., Emanuelli B., Hirschey M.D., Boucher J., Lee K.Y., Lombard D., Verdin E.M., Kahn C.R. (2011). Sirtuin-3 (Sirt3) regulates skeletal muscle metabolism and insulin signaling via altered mitochondrial oxidation and reactive oxygen species production. Proc. Natl. Acad. Sci. USA.

[132] Qiu X., Brown K., Hirschey M.D., Verdin E., Chen D. (2010). Calorie restriction reduces oxidative stress by SIRT3-mediated SOD2 activation. Cell Metab..

[133] Bauer T.M., Murphy E. (2020). Role of mitochondrial calcium and the permeability transition pore in regulating cell death. Circ. Res..

[134] Huang Y., Chi J., Wei F., Zhou Y., Cao Y., Wang Y. (2020). Mitochondrial DNA: A new predictor of diabetic kidney disease. Int. J. Endocrinol..

[135] Kaushal G.P., Chandrashekar K., Juncos L.A. (2019). Molecular interactions between reactive oxygen species and autophagy in kidney disease. Int. J. Mol. Sci..

[136] El-Daly M., Pulakazhi Venu V.K., Saifeddine M., Mihara K., Kang S., Fedak P., Alston L.A., Hirota S.A., Ding H., Triggle C.R. (2018). Hyperglycaemic impairment of PAR2-mediated vasodilation: Prevention by inhibition of aortic endothelial sodium-glucose-co-Transporter-2 and minimizing oxidative stress. Vasc. Pharmacol..

[137] Sa-Nguanmoo P., Tanajak P., Kerdphoo S., Jaiwongkam T., Pratchayasakul W., Chattipakorn N., Chattipakorn S.C. (2017). SGLT2-inhibitor and DPP-4 inhibitor improve brain function via attenuating mitochondrial dysfunction, insulin resistance, inflammation, and apoptosis in HFD-induced obese rats. Toxicol. Appl. Pharmacol..

[138] Hu Y., Huang L., Shen M., Liu Y., Liu G., Wu Y., Ding F., Ma K., Wang W., Zhang Y. (2019). Pioglitazone protects compression-mediated apoptosis in nucleus pulposus mesenchymal stem cells by suppressing oxidative stress. Oxid. Med. Cell Longev..

[139] De Blasio M.J., Huynh K., Qin C., Rosli S., Kiriazis H., Ayer A., Cemerlang N., Stocker R., Du X.J., McMullen J.R. (2015). Therapeutic targeting of oxidative stress with coenzyme Q10 counteracts exaggerated diabetic cardiomyopathy in a mouse model of diabetes with diminished PI3K(p110α) signaling. Free Radic. Biol. Med..

[140] Fink B.D., Guo D.F., Kulkarni C.A., Rahmouni K., Kerns R.J., Sivitz W.I. (2017). Metabolic effects of a mitochondrial-targeted coenzyme Q analog in high fat fed obese mice. Pharmacol. Res. Perspect..

[141] Kalinovich A.V., Mattsson C.L., Youssef M.R., Petrovic N., Ost M., Skulachev V.P., Shabalina I.G. (2016). Mitochondria-targeted dodecyltriphenylphosphonium (C12TPP) combats high-fat-diet-induced obesity in mice. Int. J. Obes..

[142] Axelrod C.L., King W.T., Davuluri G., Noland R.C., Hall J., Hull M., Dantas W.S., Zunica E.R., Alexopoulos S.J., Hoehn K.L. (2020). BAM15-mediated mitochondrial uncoupling protects against obesity and improves glycemic control. EMBO Mol. Med..

[143] Bozi L.H.M., Campos J.C., Zambelli V.O., Ferreira N.D., Ferreira J.C.B. (2020). Mitochondrially-targeted treatment strategies. Mol. Aspects Med..

[144] Anderson E.J., Lustig M.E., Boyle K.E., Woodlief T.L., Kane D.A., Lin C.T., Price J.W., Kang L., Rabinovitch P.S., Szeto H.H. (2009). Mitochondrial H_2_O_2_ emission and cellular redox state link excess fat intake to insulin resistance in both rodents and humans. J. Clin. Investig..

[145] Arkat S., Umbarkar P., Singh S., Sitasawad S.L. (2016). Mitochondrial peroxiredoxin-3 protects against hyperglycemia induced myocardial damage in diabetic cardiomyopathy. Free Radic. Biol. Med..

[146] Shen X., Zheng S., Metreveli N.S., Epstein P.N. (2006). Protection of cardiac mitochondria by overexpression of MnSOD reduces diabetic cardiomyopathy. Diabetes.

[147] Idevall-Hagren O., Tengholm A. (2020). Metabolic regulation of calcium signaling in beta cells. Semin. Cell Dev. Biol..

[148] Draznin B., Sussman K.E., Eckel R.H., Kao M., Yost T., Sherman N.A. (1988). Possible role of cytosolic free calcium concentrations in mediating insulin resistance of obesity and hyperinsulinemia. J. Clin. Investig..

[149] Miranda-Silva D., Wüst R., Conceição G., Gonçalves-Rodrigues P., Gonçalves N., Gonçalves A., Kuster D., Leite-Moreira A.F., van der Velden J., de Sousa Beleza J.M. (2020). Disturbed cardiac mitochondrial and cytosolic calcium handling in a metabolic risk-related rat model of heart failure with preserved ejection fraction. Acta Physiol..

[150] Chan K.M., Junger K.D. (1984). The effect of streptozocin-induced diabetes on the plasma membrane calcium uptake activity of rat liver. Diabetes.

[151] Leanza L., Checchetto V., Biasutto L., Rossa A., Costa R., Bachmann M., Zoratti M., Szabo I. (2019). Pharmacological modulation of mitochondrial ion channels. Br. J. Pharmacol..

[152] Tarasova N.V., Vishnyakova P.A., Logashina Y.A., Elchaninov A.V. (2019). Mitochondrial calcium uniporter structure and function in different types of muscle tissues in health and disease. Int. J. Mol. Sci..

[153] Georgiadou E., Haythorne E., Dickerson M.T., Lopez-Noriega L., Pullen T.J., da Silva Xavier G., Davis S., Martinez-Sanchez A., Semplici F., Rizzuto R. (2020). The pore-forming subunit MCU of the mitochondrial Ca^2+^ uniporter is required for normal glucose-stimulated insulin secretion in vitro and in vivo in mice. Diabetologia.

[154] Tarasov A.I., Semplici F., Ravier M.A., Bellomo E.A., Pullen T.J., Gilon P., Sekler I., Rizzuto R., Rutter G.A. (2012). The mitochondrial Ca^2+^ uniporter MCU is essential for glucose-induced ATP increases in pancreatic β-cells. PLoS ONE.

[155] Ly L.D., Ly D.D., Nguyen N.T., Kim J.H., Yoo H., Chung J., Lee M.S., Cha S.K., Park K.S. (2020). Mitochondrial Ca^2+^ uptake relieves palmitate-induced cytosolic Ca^2+^ overload in MIN6 cells. Mol. Cells.

[156] Sabatini P.V., Speckmann T., Lynn F.C. (2019). Friend and foe: β-cell Ca^2+^ signaling and the development of diabetes. Mol. Metab..

[157] Wang C.H., Wei Y.H. (2017). Role of mitochondrial dysfunction and dysregulation of Ca^2+^ homeostasis in the pathophysiology of insulin resistance and type 2 diabetes. J. Biomed. Sci..

[158] Fauconnier J., Lanner J.T., Zhang S.J., Tavi P., Bruton J.D., Katz A., Westerblad H. (2005). Insulin and inositol 1,4,5-trisphosphate trigger abnormal cytosolic Ca^2+^ transients and reveal mitochondrial Ca^2+^ handling defects in cardiomyocytes of ob/ob mice. Diabetes.

[159] Oliveira P.J., Seiça R., Coxito P.M., Rolo A.P., Palmeira C.M., Santos M.S., Moreno A.J. (2003). Enhanced permeability transition explains the reduced calcium uptake in cardiac mitochondria from streptozotocin-induced diabetic rats. FEBS Lett..

[160] Diaz-Juarez J., Suarez J., Cividini F., Scott B.T., Diemer T., Dai A., Dillmann W.H. (2016). Expression of the mitochondrial calcium uniporter in cardiac myocytes improves impaired mitochondrial calcium handling and metabolism in simulated hyperglycemia. Am. J. Physiol. Cell Physiol..

[161] Suarez J., Cividini F., Scott B.T., Lehmann K., Diaz-Juarez J., Diemer T., Dai A., Suarez J.A., Jain M., Dillmann W.H. (2018). Restoring mitochondrial calcium uniporter expression in diabetic mouse heart improves mitochondrial calcium handling and cardiac function. J. Biol. Chem..

[162] Ji L., Liu F., Jing Z., Huang Q., Zhao Y., Cao H., Li J., Yin C., Xing J., Li F. (2017). MICU1 alleviates diabetic cardiomyopathy through mitochondrial Ca^2+^—Dependent antioxidant response. Diabetes.

[163] Garrick R.A., Hall J.C. (1974). Adenosine diphosphate and calcium stimulation of respiration in mitochondria from alloxan diabetic rats. J. Cell Physiol..

[164] Wright L.E., Vecellio Reane D., Milan G., Terrin A., Di Bello G., Belligoli A., Sanna M., Foletto M., Favaretto F., Raffaello A. (2017). Increased mitochondrial calcium uniporter in adipocytes underlies mitochondrial alterations associated with insulin resistance. Am. J. Physiol. Endocrinol. Metab..

[165] Zu Y., Wan L.J., Cui S.Y., Gong Y.P., Li C.L. (2015). The mitochondrial Na(+)/Ca(2+) exchanger may reduce high glucose-induced oxidative stress and nucleotide-binding oligomerization domain receptor 3 inflammasome activation in endothelial cells. J. Geriatr. Cardiol..

[166] Leem J., Koh E.H. (2012). Interaction between mitochondria and the endoplasmic reticulum: Implications for the pathogenesis of type 2 diabetes mellitus. Exp. Diabetes Res..

[167] Thivolet C., Vial G., Cassel R., Rieusset J., Madec A.M. (2017). Reduction of endoplasmic reticulum-mitochondria interactions in beta cells from patients with type 2 diabetes. PLoS ONE.

[168] Tubbs E., Theurey P., Vial G., Bendridi N., Bravard A., Chauvin M.A., Ji-Cao J., Zoulim F., Bartosch B., Ovize M. (2014). Mitochondria-associated endoplasmic reticulum membrane (MAM) integrity is required for insulin signaling and is implicated in hepatic insulin resistance. Diabetes.

[169] Zhang E., Mohammed Al-Amily I., Mohammed S., Luan C., Asplund O., Ahmed M., Ye Y., Ben-Hail D., Soni A., Vishnu N. (2019). Preserving insulin secretion in diabetes by inhibiting VDAC1 overexpression and surface translocation in β cells. Cell Metab..

[170] Sasaki K., Donthamsetty R., Heldak M., Cho Y.E., Scott B.T., Makino A. (2012). VDAC: Old protein with new roles in diabetes. Am. J. Physiol. Cell Physiol..

[171] Zhang J., Guo Y., Ge W., Zhou X., Pan M. (2018). High glucose induces the apoptosis of HUVECs in mitochondria dependent manner by enhancing VDAC1 expression. Pharmazie.

[172] Bonora M., Patergnani S., Ramaccini D., Morciano G., Pedriali G., Kahsay A.E., Bouhamida E., Giorgi C., Wieckowski M.R., Pinton P. (2020). Physiopathology of the permeability transition pore: Molecular mechanisms in human pathology. Biomolecules.

[173] Briston T., Selwood D.L., Szabadkai G., Duchen M.R. (2019). Mitochondrial permeability transition: A molecular lesion with multiple drug targets. Trends Pharmacol. Sci..

[174] Šileikytė J., Forte M. (2019). The mitochondrial permeability transition in mitochondrial disorders. Oxid. Med. Cell Longev..

[175] Zoratti M., Szabò I. (1995). The mitochondrial permeability transition. Biochim. Biophys. Acta.

[176] Shanmughapriya S., Rajan S., Hoffman N.E., Higgins A.M., Tomar D., Nemani N., Hines K.J., Smith D.J., Eguchi A., Vallem S. (2015). SPG7 is an essential and conserved component of the mitochondrial permeability transition pore. Mol. Cell.

[177] Efimov S.V., Dubinin M.V., Kobchikova P.P., Zgadzay Y.O., Khodov I.A., Belosludtsev K.N., Klochkov V.V. (2020). Comparison of cyclosporin variants B-E based on their structural properties and activity in mitochondrial membranes. Biochem. Biophys. Res. Commun..

[178] Hurst S., Baggett A., Csordas G., Sheu S.S. (2019). SPG7 targets the m-AAA protease complex to process MCU for uniporter assembly, Ca^2+^ influx, and regulation of mitochondrial permeability transition pore opening. J. Biol. Chem..

[179] Halestrap A.P., Richardson A.P. (2015). The mitochondrial permeability transition: A current perspective on its identity and role in ischaemia/reperfusion injury. J. Mol. Cell Cardiol..

[180] Rück A., Dolder M., Wallimann T., Brdiczka D. (1998). Reconstituted adenine nucleotide translocase forms a channel for small molecules comparable to the mitochondrial permeability transition pore. FEBS Lett..

[181] Beutner G., Rück A., Riede B., Brdiczka D. (1998). Complexes between porin, hexokinase, mitochondrial creatine kinase and adenylate translocator display properties of the permeability transition pore. Implication for regulation of permeability transition by the kinases. Biochim. Biophys. Acta.

[182] Crompton M., Virji S., Ward J.M. (1998). Cyclophilin-D binds strongly to complexes of the voltage-dependent anion channel and the adenine nucleotide translocase to form the permeability transition pore. Eur. J. Biochem..

[183] Kokoszka J.E., Waymire K.G., Levy S.E., Sligh J.E., Cai J., Jones D.P., MacGregor G.R., Wallace D.C. (2004). The ADP/ATP translocator is not essential for the mitochondrial permeability transition pore. Nature.

[184] Leung A.W., Varanyuwatana P., Halestrap A.P. (2008). The mitochondrial phosphate carrier interacts with cyclophilin D and may play a key role in the permeability transition. J. Biol. Chem..

[185] Antoniel M., Jones K., Antonucci S., Spolaore B., Fogolari F., Petronilli V., Giorgio V., Carraro M., Di Lisa F., Forte M. (2018). The unique histidine in OSCP subunit of F-ATP synthase mediates inhibition of the permeability transition pore by acidic pH. EMBO Rep..

[186] Giorgio V., von Stockum S., Antoniel M., Fabbro A., Fogolari F., Forte M., Glick G.D., Petronilli V., Zoratti M., Szabó I. (2013). Dimers of mitochondrial ATP synthase form the permeability transition pore. Proc. Natl. Acad. Sci. USA.

[187] Carraro M., Giorgio V., Šileikytė J., Sartori G., Forte M., Lippe G., Zoratti M., Szabò I., Bernardi P. (2014). Channel formation by yeast F-ATP synthase and the role of dimerization in the mitochondrial permeability transition. J. Biol. Chem..

[188] von Stockum S., Giorgio V., Trevisan E., Lippe G., Glick G.D., Forte M.A., Da-Rè C., Checchetto V., Mazzotta G., Costa R. (2015). F-ATPase of drosophila melanogaster forms 53-picosiemen (53-pS) channels responsible for mitochondrial Ca^2+^-induced Ca^2+^ release. J. Biol. Chem..

[189] Carraro M., Checchetto V., Sartori G., Kucharczyk R., di Rago J.P., Minervini G., Franchin C., Arrigoni G., Giorgio V., Petronilli V. (2018). High-conductance channel formation in yeast mitochondria is mediated by F-ATP synthase e and g subunits. Cell Physiol. Biochem..

[190] Mnatsakanyan N., Llaguno M.C., Yang Y., Yan Y., Weber J., Sigworth F.J., Jonas E.A. (2019). A mitochondrial megachannel resides in monomeric F1FO ATP synthase. Nat. Commun..

[191] Bonora M., Morganti C., Morciano G., Pedriali G., Lebiedzinska-Arciszewska M., Aquila G., Giorgi C., Rizzo P., Campo G., Ferrari R. (2017). Mitochondrial permeability transition involves dissociation of F1FO ATP synthase dimers and C-ring conformation. EMBO Rep..

[192] Alavian K.N., Beutner G., Lazrove E., Sacchetti S., Park H.A., Licznerski P., Li H., Nabili P., Hockensmith K., Graham M. (2014). A An uncoupling channel within the c-subunit ring of the F1FO ATP synthase is the mitochondrial permeability transition pore. Proc. Natl. Acad. Sci. USA.

[193] Bernardi P., Rasola A., Forte M., Lippe G. (2015). The mitochondrial permeability transition pore: Channel formation by F-ATP synthase, integration in signal transduction, and role in pathophysiology. Physiol. Rev..

[194] Zhou W., Marinelli F., Nief C., Faraldo-Gómez J.D. (2017). Atomistic simulations indicate the c-subunit ring of the F1Fo ATP synthase is not the mitochondrial permeability transition pore. eLife.

[195] Neginskaya M.A., Solesio M.E., Berezhnaya E.V., Amodeo G.F., Mnatsakanyan N., Jonas E.A., Pavlov E.V. (2019). ATP synthase C-subunit-deficient mitochondria have a small cyclosporine A-sensitive channel, but lack the permeability transition pore. Cell Rep..

[196] Karch J., Bround M.J., Khalil H., Sargent M.A., Latchman N., Terada N., Peixoto P.M., Molkentin J.D. (2019). Inhibition of mitochondrial permeability transition by deletion of the ANT family and CypD. Sci. Adv..

[197] Baines C.P., Gutiérrez-Aguilar M. (2020). The mitochondrial permeability transition pore: Is it formed by the ATP synthase, adenine nucleotide translocators or both?. Biochim. Biophys. Acta Bioenerg..

[198] Agafonov A., Gritsenko E., Belosludtsev K., Kovalev A., Gateau-Roesch O., Saris N.E., Mironova G.D. (2003). A permeability transition in liposomes induced by the formation of Ca^2+^/palmitic acid complexes. Biochim. Biophys. Acta.

[199] Belosludtsev K.N., Belosludtseva N.V., Agafonov A.V., Astashev M.E., Kazakov A.S., Saris N.E., Mironova G.D. (2014). Ca^2+^-dependent permeabilization of mitochondria and liposomes by palmitic and oleic acids: A comparative study. Biochim. Biophys. Acta.

[200] Mironova G.D., Saris N.E., Belosludtseva N.V., Agafonov A.V., Elantsev A.B., Belosludtsev K.N. (2015). Involvement of palmitate/Ca^2+^(Sr^2+^)-induced pore in the cycling of ions across the mitochondrial membrane. Biochim. Biophys. Acta.

[201] Lablanche S., Cottet-Rousselle C., Lamarche F., Benhamou P.Y., Halimi S., Leverve X., Fontaine E. (2011). Protection of pancreatic INS-1 β-cells from glucose- and fructose-induced cell death by inhibiting mitochondrial permeability transition with cyclosporin A or metformin. Cell Death Dis..

[202] Lablanche S., Cottet-Rousselle C., Argaud L., Laporte C., Lamarche F., Richard M.J., Berney T., Benhamou P.Y., Fontaine E. (2015). Respective effects of oxygen and energy substrate deprivation on beta cell viability. Biochim. Biophys. Acta.

[203] Fujimoto K., Chen Y., Polonsky K.S., Dorn G.W. (2010). Targeting cyclophilin D and the mitochondrial permeability transition enhances beta-cell survival and prevents diabetes in Pdx1 deficiency. Proc. Natl. Acad. Sci. USA.

[204] Monaco C., Hughes M.C., Ramos S.V., Varah N.E., Lamberz C., Rahman F.A., McGlory C., Tarnopolsky M.A., Krause M.P., Laham R. (2018). Altered mitochondrial bioenergetics and ultrastructure in the skeletal muscle of young adults with type 1 diabetes. Diabetologia.

[205] Lumini-Oliveira J., Ascensão A., Pereira C.V., Magalhães S., Marques F., Oliveira P.J., Magalhães J. (2010). Long-term hyperglycaemia decreases gastrocnemius susceptibility to permeability transition. Eur. J. Clin. Investig..

[206] Couturier K., Hininger I., Poulet L., Anderson R.A., Roussel A.M., Canini F., Batandier C. (2016). Cinnamon intake alleviates the combined effects of dietary-induced insulin resistance and acute stress on brain mitochondria. J. Nutr. Biochem..

[207] Moreira P.I., Santos M.S., Moreno A.M., Proença T., Seiça R., Oliveira C.R. (2004). Effect of streptozotocin-induced diabetes on rat brain mitochondria. J. Neuroendocrinol..

[208] Oliveira P.J., Esteves T.C., Seiça R., Moreno A.J., Santos M.S. (2004). Calcium-dependent mitochondrial permeability transition is augmented in the kidney of Goto-Kakizaki diabetic rat. Diabetes Metab. Res. Rev..

[209] Williamson C.L., Dabkowski E.R., Baseler W.A., Croston T.L., Always S.E., Hollander J.M. (2010). Enhanced apoptotic propensity in diabetic cardiac mitochondria: Influence of subcellular spatial location. Am. J. Physiol. Heart Circ. Physiol..

[210] Yan S., Du F., Wu L., Zhang Z., Zhong C., Yu Q., Wang Y., Lue L.F., Walker D.G., Douglas J.T. (2016). F1F0 ATP synthase-cyclophilin D interaction contributes to diabetes-induced synaptic dysfunction and cognitive decline. Diabetes.

[211] Lindblom R., Higgins G.C., Nguyen T.V., Arnstein M., Henstridge D.C., Granata C., Snelson M., Thallas-Bonke V., Cooper M.E., Forbes J.M. (2020). Delineating a role for the mitochondrial permeability transition pore in diabetic kidney disease by targeting cyclophilin D. Clin. Sci..

[212] Dabkowski E.R., Baseler W.A., Williamson C.L., Powell M., Razunguzwa T.T., Frisbee J.C., Hollander J.M. (2010). Mitochondrial dysfunction in the type 2 diabetic heart is associated with alterations in spatially distinct mitochondrial proteomes. Am. J. Physiol. Heart Circ. Physiol..

[213] Sparks L.M., Gemmink A., Phielix E., Bosma M., Schaart G., Moonen-Kornips E., Jörgensen J.A., Nascimento E.B., Hesselink M.K., Schrauwen P. (2016). ANT1-mediated fatty acid-induced uncoupling as a target for improving myocellular insulin sensitivity. Diabetologia.

[214] Zhang L., Yu C., Vasquez F.E., Galeva N., Onyango I., Swerdlow R.H., Dobrowsky R.T. (2010). Hyperglycemia alters the schwann cell mitochondrial proteome and decreases coupled respiration in the absence of superoxide production. J. Proteome Res..

[215] Andelova N., Waczulikova I., Talian I., Sykora M., Ferko M. (2020). mPTP proteins regulated by streptozotocin-induced diabetes mellitus are effectively involved in the processes of maintaining myocardial metabolic adaptation. Int. J. Mol. Sci..

[216] Munusamy S., Saba H., Mitchell T., Megyesi J.K., Brock R.W., Macmillan-Crow L.A. (2009). Alteration of renal respiratory Complex-III during experimental type-1 diabetes. BMC Endocr. Dis..

[217] Sloan R.C., Moukdar F., Frasier C.R., Patel H.D., Bostian P.A., Lust R.M., Brown D.A. (2012). Mitochondrial permeability transition in the diabetic heart: Contributions of thiol redox state and mitochondrial calcium to augmented reperfusion injury. J. Mol. Cell Cardiol..

[218] Taddeo E.P., Laker R.C., Breen D.S., Akhtar Y.N., Kenwood B.M., Liao J.A., Zhang M., Fazakerley D.J., Tomsig J.L., Harris T.E. (2013). Opening of the mitochondrial permeability transition pore links mitochondrial dysfunction to insulin resistance in skeletal muscle. Mol. Metab..

[219] Li P.A., Uchino H., Elmér E., Siesjö B.K. (1997). Amelioration by cyclosporin A of brain damage following 5 or 10 min of ischemia in rats subjected to preischemic hyperglycemia. Brain Res..

[220] Qi R., Wang D., Xing L., Wu Z. (2018). Cyclosporin A inhibits mitochondrial biogenesis in Hep G2 cells. Biochem. Biophys. Res. Commun..

[221] Koshkin V., Bikopoulos G., Chan C.B., Wheeler M.B. (2004). The characterization of mitochondrial permeability transition in clonal pancreatic beta-cells. Multiple modes and regulation. J. Biol. Chem..

[222] Lu A., Chu C., Mulvihill E., Wang R., Liang W. (2019). ATP-sensitive K^+^ channels and mitochondrial permeability transition pore mediate effects of hydrogen sulfide on cytosolic Ca^2+^ homeostasis and insulin secretion in β-cells. Pflugers Arch..

[223] Kim J.W., Yang J.H., Park H.S., Sun C., Lee S.H., Cho J.H., Yang C.W., Yoon K.H. (2009). Rosiglitazone protects the pancreatic beta-cell death induced by cyclosporine A. Biochem. Biophys. Res. Commun..

[224] Taddeo E.P., Alsabeeh N., Baghdasarian S., Wikstrom J.D., Ritou E., Sereda S., Erion K., Li J., Stiles L., Abdulla M. (2020). Mitochondrial proton leak regulated by cyclophilin D elevates insulin secretion in islets at nonstimulatory glucose levels. Diabetes.

[225] Bugliani M., Masini M., Liechti R., Marselli L., Xenarios I., Boggi U., Filipponi F., Masiello P., Marchetti P. (2009). The direct effects of tacrolimus and cyclosporin A on isolated human islets: A functional, survival and gene expression study. Islets.

[226] Ebihara K., Fukunaga K., Matsumoto K., Shichiri M., Miyamoto E. (1996). Cyclosporin A stimulation of glucose-induced insulin secretion in MIN6 cells. Endocrinology.

[227] Riojas-Hernández A., Bernal-Ramírez J., Rodríguez-Mier D., Morales-Marroquín F.E., Domínguez-Barragán E.M., Borja-Villa C., Rivera-Álvarez I., García-Rivas G., Altamirano J., García N. (2015). Enhanced oxidative stress sensitizes the mitochondrial permeability transition pore to opening in heart from Zucker Fa/fa rats with type 2 diabetes. Life Sci..

[228] Itoh T., Kouzu H., Miki T., Tanno M., Kuno A., Sato T., Sunaga D., Murase H., Miura T. (2012). Cytoprotective regulation of the mitochondrial permeability transition pore is impaired in type 2 diabetic Goto-Kakizaki rat hearts. J. Mol. Cell Cardiol..

[229] Boudina S., Sena S., Theobald H., Sheng X., Wright J.J., Hu X.X., Aziz S., Johnson J.I., Bugger H., Zaha V.G. (2007). Mitochondrial energetics in the heart in obesity-related diabetes: Direct evidence for increased uncoupled respiration and activation of uncoupling proteins. Diabetes.

[230] Baseler W.A., Dabkowski E.R., Williamson C.L., Croston T.L., Thapa D., Powell M.J., Razunguzwa T.T., Hollander J.M. (2011). Proteomic alterations of distinct mitochondrial subpopulations in the type 1 diabetic heart: Contribution of protein import dysfunction. Am. J. Physiol. Regul. Integr. Comp. Physiol..

[231] Lumini-Oliveira J., Magalhães J., Pereira C.V., Moreira A.C., Oliveira P.J., Ascensão A. (2011). Endurance training reverts heart mitochondrial dysfunction, permeability transition and apoptotic signaling in long-term severe hyperglycemia. Mitochondrion.

[232] Najafi M., Farajnia S., Mohammadi M., Badalzadeh R., Ahmadi Asl N., Baradaran B., Amani M. (2014). Inhibition of mitochondrial permeability transition pore restores the cardioprotection by postconditioning in diabetic hearts. J. Diabetes Metab. Disord..

[233] Huhn R., Heinen A., Hollmann M.W., Schlack W., Preckel B., Weber N.C. (2010). Cyclosporine A administered during reperfusion fails to restore cardioprotection in prediabetic Zucker obese rats in vivo. Nutr. Metab. Cardiovasc. Dis..

[234] Morrow R.M., Picard M., Derbeneva O., Leipzig J., McManus M.J., Gouspillou G., Barbat-Artigas S., Dos Santos C., Hepple R.T., Murdock D.G. (2017). Mitochondrial energy deficiency leads to hyperproliferation of skeletal muscle mitochondria and enhanced insulin sensitivity. Proc. Natl. Acad. Sci. USA.

[235] Moreira P.I., Santos M.S., Moreno A.M., Seiça R., Oliveira C.R. (2003). Increased vulnerability of brain mitochondria in diabetic (Goto-Kakizaki) rats with aging and amyloid-beta exposure. Diabetes.

[236] Krügel K., Wurm A., Pannicke T., Hollborn M., Karl A., Wiedemann P., Reichenbach A., Kohen L., Bringmann A. (2011). Involvement of oxidative stress and mitochondrial dysfunction in the osmotic swelling of retinal glial cells from diabetic rats. Exp. Eye Res..

[237] Klawitter J., Pennington A., Klawitter J., Thurman J.M., Christians U. (2017). Mitochondrial cyclophilin D ablation is associated with the activation of Akt/p70S6K pathway in the mouse kidney. Sci. Rep..

[238] Kristal B.S., Matsuda M., Yu B.P. (1996). Abnormalities in the mitochondrial permeability transition in diabetic rats. Biochem. Biophys. Res. Commun..

[239] Rieusset J., Fauconnier J., Paillard M., Belaidi E., Tubbs E., Chauvin M.A., Durand A., Bravard A., Teixeira G., Bartosch B. (2016). Disruption of calcium transfer from ER to mitochondria links alterations of mitochondria-associated ER membrane integrity to hepatic insulin resistance. Diabetologia.

[240] Sobel D.O., Henzke A., Abbassi V. (2010). Cyclosporin and methotrexate therapy induces remission in type 1 diabetes mellitus. Acta Diabetol..

[241] Devalaraja-Narashimha K., Diener A.M., Padanilam B.J. (2011). Cyclophilin D deficiency prevents diet-induced obesity in mice. FEBS Lett..

[242] Guigas B., Detaille D., Chauvin C., Batandier C., De Oliveira F., Fontaine E., Leverve X. (2004). Metformin inhibits mitochondrial permeability transition and cell death: A pharmacological in vitro study. Biochem. J..

[243] El-Mir M.Y., Detaille D., Villanueva G.R., Delgado-Esteban M., Guigas B., Attia S., Fontaine E., Almeida A., Leverve X. (2008). Neuroprotective role of antidiabetic drug metformin against apoptotic cell death in primary cortical neurons. J. Mol. Neurosci..

[244] Carvalho C., Correia S., Santos M.S., Seiça R., Oliveira C.R., Moreira P.I. (2008). Metformin promotes isolated rat liver mitochondria impairment. Mol. Cell Biochem..

[245] Masubuchi Y., Kano S., Horie T. (2006). Mitochondrial permeability transition as a potential determinant of hepatotoxicity of antidiabetic thiazolidinediones. Toxicology.

[246] Segawa M., Sekine S., Sato T., Ito K. (2018). Increased susceptibility to troglitazone-induced mitochondrial permeability transition in type 2 diabetes mellitus model rat. J. Toxicol. Sci..

[247] Yang J.T., Qian L.B., Zhang F.J., Wang J., Ai H., Tang L.H., Wang H.P. (2015). Cardioprotective effects of luteolin on ischemia/reperfusion injury in diabetic rats are modulated by eNOS and the mitochondrial permeability transition pathway. J. Cardiovasc. Pharmacol..

[248] Zhong Y., Jin J., Liu P., Song Y., Zhang H., Sheng L., Zhou H., Jiang B. (2020). Berberine attenuates hyperglycemia by inhibiting the hepatic glucagon pathway in diabetic mice. Oxid. Med. Cell Longev..

[249] Pereira C.V., Machado N.G., Oliveira P.J. (2008). Mechanisms of berberine (natural yellow 18)-induced mitochondrial dysfunction: Interaction with the adenine nucleotide translocator. Toxicol. Sci..

